# Altered splicing of ATG16‐L1 mediates acquired resistance to tyrosine kinase inhibitors of EGFR by blocking autophagy in non‐small cell lung cancer

**DOI:** 10.1002/1878-0261.13229

**Published:** 2022-08-30

**Authors:** Anne‐Sophie Hatat, Clara Benoit‐Pilven, Amélie Pucciarelli, Florence de Fraipont, Lucie Lamothe, Pascal Perron, Amandine Rey, Matteo Giaj Levra, Anne‐Claire Toffart, Didier Auboeuf, Beatrice Eymin, Sylvie Gazzeri

**Affiliations:** ^1^ Team “RNA Splicing, Cell Signaling and Response to Therapies”, Institute for Advanced Biosciences (IAB) INSERM U1209, CNRS UMR 5309, Grenoble Alpes University France; ^2^ Laboratory of Biology and Modelling of the Cell Univ Lyon, ENS de Lyon, Univ Claude Bernard, CNRS UMR 5239, INSERM U1210 Lyon France; ^3^ Molecular Genetic Unit Grenoble‐Alpes University Hospital France; ^4^ Thoracic Oncology Unit Grenoble‐Alpes University Hospital France

**Keywords:** ATG16‐L1, autophagy, EGFR‐TKI, non‐small cell lung cancer, resistance

## Abstract

Despite the initial efficacy of using tyrosine kinase inhibitors of epidermal growth factor receptors (EGFR‐TKIs) for treating patients with non‐small cell lung cancer (NSCLC), resistance inevitably develops. Recent studies highlight a link between alternative splicing and cancer drug response. Therefore, we aimed to identify deregulated splicing events that play a role in resistance to EGFR‐TKI. By using RNA sequencing, reverse‐transcription PCR (RT‐PCR), and RNA interference, we showed that overexpression of a splice variant of the autophagic gene *ATG16‐L1* that retains exon 8 and encodes the β‐isoform of autophagy‐related protein 16‐1 (ATG16‐L1 β) concurs acquired resistance to EGFR‐TKI in NSCLC cells. Using matched biopsies, we found increased levels of ATG16‐L1 β at the time of progression in 3 of 11 NSCLC patients treated with EGFR‐TKI. Mechanistically, gefitinib‐induced autophagy was impaired in resistant cells that accumulated ATG16‐L1 β. Neutralization of ATG16‐L1 β restored autophagy in response to gefitinib, induced apoptosis, and inhibited the growth of *in ovo* tumor xenografts. Conversely, overexpression of ATG16‐L1 β in parental sensitive cells prevented gefitinib‐induced autophagy and increased cell survival. These results support a role of defective autophagy in acquired resistance to EGFR‐TKIs and identify splicing regulation of *ATG16‐L1* as a therapeutic vulnerability that could be explored for improving EGFR‐targeted cancer therapy.

## Graphical abstract

Chronic exposure of lung tumors to EGFR‐TKI induces deregulated splicing of ATG16‐L1 in favor of the β isoform that retains exon 8. This blocks the induction of autophagy in response to EGFR‐TKI and inhibits apoptosis thereby inducing resistance. Hence, correcting ATG16‐L1 splicing could represent a new therapeutic approach to overcome resistance to EGFR‐TKI.

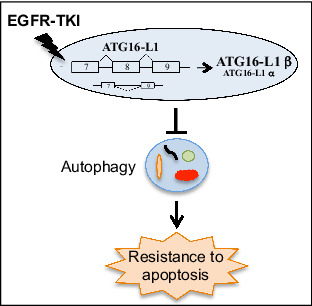

**Abbreviations**
NSCLC: non‐small cell lung cancer
SCLC: small cell lung cancer
EGFR: epidermal growth factor receptor
TKI: tyrosine kinase inhibitor
HER‐2: human epidermal growth factor receptor 2
SR protein: Ser/Arg‐rich protein
VEGF‐A: vascular endothelial growth factor A
VEGFR1: vascular endothelial growth factor receptor 1
ATG16‐L1: autophagy‐related 16 like 1
LC3: microtubule‐associated protein 1A/1B‐light chain 3
ALK: anaplastic lymphoma kinase
PI3KCA: phosphatidylinositol‐4,5‐biphosphate 3‐kinase catalytic subunit alpha
FGFR1: fibroblast growth factor receptor 1
SQSTM1: sequestosome 1
LC3: microtubule‐associated protein 1A/1B‐light chain 3RT
GAPDH: glyceraldehyde‐3‐phosphate deshydrogenase
GFP: green fluorescent protein
PARP: poly(ADP‐ribose) polymerase 1
mTOR: mechanistic target of rapamycin kinase
RT: reverse transcription
PCR: polymerase chain reaction
FISH: fluorescent *in situ* hybridization
FACS: fluorescence‐activated cell sorting
PE: phycoerythrin
FFPE: formalin‐fixed paraffin‐embedded
CAM: chicken chorioallantoid membranes
PSI: percentage spliced‐in
SD: standard deviation
ES: exon skipping
AAS: alternative acceptor site
ADS: alternative donor site
MEE: mutually exclusive exons
MES: multiexon skipping

## 1 Introduction

In the past decade, the epidermal growth factor receptor (EGFR) has become an important therapeutic target for patients with lung cancer [1]. EGFR tyrosine kinase inhibitors (TKIs) have greatly improved the prognosis of non‐small cell lung cancer (NSCLC) patients with EGFR‐TKI sensitizing mutations over traditional platinum‐based chemotherapy [2, 3, 4, 5, 6]. However, despite important responses in previously intractable cases, first‐generation (*e.g*., gefitinib and erlotinib) and second‐generation (*e.g*., dacomitinib and afatinib) EGFR‐TKIs provide in most instances only a few additional months of tumor‐free survival because of rapid development of resistance. The most common resistance mechanism results from the appearance of the secondary T790M EGFR mutation (50% of cases) [7, 8, 9]. Third‐generation EGFR‐TKIs (such as osimertinib) that selectively target EGFR mutations including the T790M have demonstrated robust clinical activity, as both first‐ and second‐line treatments [10, 11, 12, 13, 14], but again, patients inevitably develop secondary resistance. Diverse mechanisms of EGFR‐independent resistance to EGFR‐TKIs have been identified that mainly include activation of bypassing signaling pathways (*e.g*., MET or HER2) and histological or phenotypical transformation [15, 16]. However, these mechanisms are not fully understood, and the treatment of T790M‐negative NSCLC still remains an area of unmet medical need. Based on the inevitable evolution of lung tumors under EGFR‐TKI treatment, it is critical to characterize the mechanisms underlying drug resistance in order to adapt the therapeutic strategy.

Pre‐mRNA alternative splicing is a key molecular mechanism that increases the functional diversity of the eukaryotic proteomes. Genome‐wide studies have long revealed the existence of cancer‐associated splicing patterns [17, 18]. Both mutations in cis‐acting splicing regulatory elements and alterations in the expression and/or activity of splicing factors markedly affect the splicing profile of many cancer‐associated genes. For instance, we and others have previously reported aberrant expression of splicing factors of the SR (Ser/Arg‐rich) family in lung cancer, and have identified some splicing events that correlate with disease progression [19, 20, 21]. Protein isoforms produced from mRNA splicing variants may also influence the clinical response to cancer treatment. In head and neck tumors, the EGFR variant III isoform (EGFRvIII) mediates resistance to cetuximab [22]. In NSCLC, we recently demonstrated that aberrant splicing of VEGF‐A or VEGFR1 promotes tumor escape from anti‐angiogenic therapies [23, 24]. However, although these studies support a role of splicing in drug response, this is still an emerging field with only a few examples being described up to now, especially in the context of acquired resistance. Deciphering whether deregulated alternative splicing contributes to resistance to cancer treatment could offer new therapeutic strategies.

Autophagy is an essential process for tissue homeostasis that is responsible for degrading long‐lived proteins and maintaining amino acid pools during stress conditions, such as chronic starvation. Macroautophagy, the best‐characterized type of autophagy, involves the sequestration of cytosol and organelles into the so‐called autophagosome, which is subsequently delivered to the lysosome for bulk degradation. Autophagosome formation and breakdown are regulated by autophagy‐related (ATG) genes. ATG16‐L1 is a critical component of autophagy as it contributes to the elongation and expansion of the phagophore [25]. ATG16‐L1 has multiple isoforms produced by alternative splicing [26]. In mammals ATG16‐L1 is mainly expressed as α‐ and β‐isoforms that distinguish by the addition of a small nucleotide sequence only in the β‐isoform. It has been shown that the two isoforms display distinct functions in membrane binding and LC3B lipidation in autophagy‐related processes according to the cellular context [27]. In general, autophagy is considered a survival process, but in some conditions, autophagy can induce cell death [28, 29]. In cancer treatment, autophagy appears to play this dual role, being highly contextual and dependent on many factors including tumor origin and treatment type [30, 31, 32, 33]. EGFR‐TKIs have been reported to induce autophagy in NSCLC cell lines [34, 35, 36, 37, 38], but whether autophagy contributes to the death or survival of treated cells remains a subject of debate.

The aim of this study was to investigate whether deregulation of alternative splicing plays a role in acquired resistance to EGFR‐TKIs. Our results show upregulation of ATG16‐L1 β splicing isoform in NSCLC with acquired resistance to EGFR‐TKIs. Notably, we show that neutralization of ATG16‐L1 β restores EGFR‐TKIs‐induced apoptosis in resistant cells through induction of autophagy and limits tumor growth.

## 2 Materials and methods

### 2.1 Cell lines, cell culture, and reagents

PC9 cells have the Del19 (Δ746–750) EGFR activating mutation and are TKI‐sensitive. They were originally provided by Dr. A. Gazdar and recently authenticated by DNA STR profiling (ATCC cell line Authentication Service, LGC standards, Molsheim, France). The gefitinib‐ and dacomitinib‐resistant cell lines were established in a manner similar to previously described methods [39]. Briefly, TKI‐sensitive cells were exposed to increasing amounts of gefitinib (from 10 to 90 nm, corresponding to IC_30_ to IC_60_ in parental cells) or dacomitinib (from 1 to 10 nm, corresponding to IC_30_ to IC_60_ in parental cells) over 7 months until cells displayed near‐normal growth kinetics. Resistant clones (PC9 GR, PC9 DR) were then isolated by limited dilution in 96‐well plates with continuous exposure to 90 nm gefitinib or 10 nm dacomitinib. The same protocol was applied to generate HCC827 GR clones from sensitive parental HCC827 cells cultured with 100–1000 nm gefitinib. Further study was performed on 3 PC9 GR (GR1, GR3, GR4), 2 PC9 DR (DR1, DR2), and 2 HCC827 GR (GR1, GR2) clones that did not carry common mechanisms of resistance (T790M EGFR, KRAS, NRAS, BRAF or PIK3CA mutation, FGFR1, PI3KCA or MET amplification, ALK or ROS1 rearrangement and SCLC phenotype) as detected by RT‐PCR, pyrosequencing and FISH analyses. Cell viability assays of resistant clones were performed in 96‐well plates using Cell Titer 96®Aqueous One Solution cell proliferation Assay (Promega, Charbonnières les Bains, France) to confirm gefitinib or dacomitinib resistance. IC50 values were calculated using graphpad prism software (GraphPad Software Inc., San Diego, CA, USA). These clones maintained resistance even after withdrawal of the EGFR‐TKI from the culture media for over 16 weeks. Clones were therefore cultured without drugs, and their resistance to EGFR‐TKI was examined periodically. The absence of mycoplasma was checked every 6 months (MycoAlert™ mycoplasma detection kit, Lonza, Ozyme, Saint‐Cyr L'Ecole, France). All cell lines were cultured in RPMI 1640 + GlutaMAX medium (Gibco, Cergy Pontoise, France) supplemented with 10% fetal calf serum (Dutcher, Issy‐les‐Moulineaux, France) and kept at 37 °C in a humidified incubator with 5% CO_2_. Gefitinib, dacomitinib, bafilomycin A1, wortmannin, and rapamycin were purchased from Selleckchem (Souffelweyersheim, France).

### 2.2 Tumor samples

Eleven patients with paired samples (at the time of diagnosis, and after progression under EGFR‐TKI treatment) were included in this study. Tumor specimens were obtained from the Grenoble‐Alpes University Hospital (La Tronche, France) under institutional review board–approved studies. The study methodologies conformed to the standards set by the Declaration of Helsinki. Study approval was obtained on January 1^st^ of 2019 by the Clinical Research and Innovation Office of Grenoble‐Alpes University Hospital. According to French law, there is an IRB waiver for this kind of research. An information letter was sent to each living patient providing him the opportunity to refuse study participation. All NSCLC samples underwent standard pathological examination. The presence of an EGFR mutation in each tumor sample was studied as described previously [40]. Patients treated with EGFR‐TKI exclusively received first‐ or second‐generation TKI (*e.g*., tarceva, iressa, or giotrif; Table S1).

### 2.3 cDNA constructs and lentiviral transduction

Full‐length ATG16‐L1 cDNA that retains exon 8 (ATG16‐L1‐203 according to Ensembl) was synthesized (GENEWIZ, Paris, France) and cloned in fusion with GFP in the N‐terminus into pSico R GFP vector with CAG promoter by Gibson assembly. Lentivirus was produced by co‐transfecting pC57GPBEB GagPol MLV, pSUSVSVG, and pSico R GFP or pSico R GFP ATG16‐L1 Ex8 using lipofectamine 2000 (Invitrogen, ThermoFisher Scientific, Courtaboeuf, France) in HEK293 FT cells plated in 6‐well plates at 50% confluency. The medium was changed 4 h later. The viral supernatant was collected after 72 h and was filtered with 0.45 μm filters. PC9 cells were plated in petri dishes so as to achieve 70% confluency on the day of infection. The filtered supernatant was directly used to infect the cells. The medium was changed 4 h after infection. After 4 days of culture in a fresh medium without lentiviral particules, cells were FACS sorted (Aria cell sorter 2000, BD, Le pont de Claix, France) based on the level of expression of GFP‐tagged ATG16‐L1, and stable cell lines were amplified for further use.

### 2.4 Transfection of siRNA oligonucleotides

Transfection was performed using JetPrime reagent (Ozyme, Saint‐Quentin‐en‐Yvelines, France) according to the manufacturer's instructions, and cells were analyzed 72 h post‐transfection. The sequences designed to specifically target human exon 8 of ATG16‐L1 and ex7‐9 junction of ATG16‐L1 RNAs were as follows: ATG16‐L1 ex8 (1) 5′‐CUGGAUUCUAUCACUAAUAUC‐3′; ATG16‐L1 ex8 (2) 5′‐CUGGAGGCCUUCUGGAUUCUA‐3′; ATG16‐L1 ex7‐9 5′‐AGCAGCCACGAGACGCUCUGUCUC‐3′. For all experiments, the mismatch siRNA oligonucleotide used as a control was: 5′‐UCGGCUCUUACGCAUUCAA‐3′.

### 2.5 RNA sequencing

Three biological replicate RNA samples from PC9‐sensitive cells and from three distinct clones resistant to gefitinib (PC9 GR1/GR3/GR4) were prepared using the NucleoSpin RNA isolation kit from Macherey‐Nagel (Hoerdt, France). Library preparation was performed using Illumina's Stranded mRNA Sample Prep kit. Reads generated with an Illumina HiSeq 2500 platform (100 bp Paired End) were mapped and filtered using TopHat2 (2.0.13), Samtools (0.1.19), and PrinSeq (0.20.4). Splicing analysis was performed using FARLINE, a freely available computational program (http://kissplice.prabi.fr/pipeline_ks_farline) that quantifies alternative splicing variations [41]. The PSI value corresponding to exon inclusion rate was used to determine the sets of exons that are differentially spliced when comparing resistant to control‐sensitive cell clones. Exons are defined as differentially regulated if the PSI variation (deltaPSI) is greater than 10% or lower than −10%, with *P*‐values <0.05, as compared to the corresponding control [41]. Exons were defined using FASTERDB (http://fasterdb.ens‐lyon.fr/faster/home.pl). RNA‐seq datasets are available at https://www.ncbi.nlm.nih.gov/geo/query/acc.cgi?acc=GSE146259 (secure token to be used by reviewers: gvixiyqorxmxfar).

### 2.6 RNA extraction and RT‐PCR

Total RNA was extracted from FFPE human samples using the QIAamp DNA FFPE tissue kit without an RNase step (Qiagen, Courtaboeuf, France). Additionally, total RNA was extracted from cell lines using the NucleoSpin RNA isolation kit (Macherey‐Nagel) according to the manufacturer's protocol. Reverse transcription was performed using iScript RT supermix (Biorad, Marnes‐La‐Coquette, France). PCR was carried out using GoTaq (Promega). The primers used in this study are listed in Table S2. PCR products were separated on 2% agarose gels and imaged using a Vilber device. Densitometric quantification of PCR products was performed using the imagej software (NIH, Bethesda, MD, USA) in order to evaluate differential splicing events in either sensitive (PC9) or resistant (PC9 GR1/GR3/GR4) cells that had been treated or not. Isoform switching was defined by the relative enrichment of the long isoform levels compared with the short isoform levels [long isoform/(long+short isoforms)] and annotated as percent spliced‐in (PSI) in each condition.

### 2.7 Apoptosis detection

Apoptosis was evaluated with the PE‐conjugated monoclonal active caspase‐3 antibody apoptosis kit (BD Biosciences, Pharmingen, Le Pont de Claix, France), according to the manufacturer's instructions, and analyzed by fluorescence‐activated cell sorting (FACS).

### 2.8 Immunoblotting

Whole‐cell extract was performed as previously described [42], and immunoblots were carried out using the following antibodies: anti‐GAPDH, anti‐actin, anti‐GFP and anti‐SQSTM1 (Santa‐Cruz Biotechnology, Cliniscience, Nanterre, France), anti‐LC3B(D11), anti‐ATG16‐L1 (D6D5), anti‐4E‐BP1 (53H11), and anti‐p4E‐BP1 (Thr37/44) (Cell Signaling Technology, Ozyme, Saint‐Cyr‐L'Ecole, France). LC3‐I and LC3‐II intensities were quantified using the imagej software, and LC3‐II/LC3‐I ratios were determined in each condition. Relative LC3‐II/LC3‐I ratios were represented as fold‐increase relative to untreated cells, which were arbitrarily assigned to 1.

### 2.9 Measurement of autophagic flux

FUW mCherry‐GFP‐LC3 was a gift from Anne Brunet (Addgene plasmid #110060, Watertown, MA, USA) [43]. Cells were transiently transfected with the plasmid using Lipofectamine 2000 (Invitrogen, ThermoFisher Scientific) for 7 h and then replaced in a fresh medium. Following 24 h of transfection, cells were treated or not with rapamycin (1 μm) or Gefitinib (0.1 μm) for 2 h, fixed with 5% formaldehyde for 15 min, and permeabilized with 0.5% Triton X‐100 for 10 min. Nuclei were counterstained with Hoechst 33342. Cells were visualized with an Axioimager microscope (Carl Zeiss, Jena, Germany) and the AxioVision^®^ software at a 64× magnification and processed with imagej software. Autophagic flux was determined by the presence of yellow puncta (the overlap of red mCherry and green GFP fluorescence signals), and red puncta (mCherry, GFP signal is quenched in the acidic environment of lysosomes).

### 2.10 Indirect immunofluorescence

Cells were fixed with 2% paraformaldehyde in PBS for 10 min at 4 °C and permeabilized with acetone for 10 min at room temperature. Blocking was performed in 5% BSA, and primary antibodies were incubated overnight at 4 °C. Anti‐LC3B(D11) was from Cell Signaling Technology (Ozyme). After incubation for 1 h with conjugated secondary antibodies, cell nuclei were counterstained with Hoechst. Cells were visualized with an Axioimager microscope (Carl Zeiss) and the axiovision ^®^ software at a 100× magnification and processed with imagej software. Autophagy was measured by visual counting of LC3 punctua (with 50 randomly selected cells for each condition).

### 2.11 In ovo growth assay

According to French legislation, no ethical approval is needed for scientific experimentations using oviparous embryos (Decree number 2013–118 February 1, 2013; art. R‐214‐88). Chick embryo tumor growth assays (Inovotion, La Tronche, France) were performed using fertilized white leghorn eggs (SFPA) that were incubated at 38 °C with 50% relative humidity for 9 days. The chorioallantoic membrane (CAM) was dropped by drilling a small hole through the eggshell into the air sac, and a 1‐cm^2^ window was cut in the eggshell above the chorioallantoic membrane. PC9 GR4 cells were transfected with mismatch (control) or ATG16‐L1 exon8 siRNAs. Cells were trypsinized after 24 h, and an inoculum of 2 × 10^6^ cells was loaded onto the CAM of each egg (E9), and eggs were returned to the incubator. Eggs were randomly allocated into four groups: mismatch (mis) (control) + vehicle; mis + gefitinib; siEx8 + vehicle; or siEx8 + gefitinib. At least 21 eggs were used for each group. Eggs were treated with gefitinib (150 μm) or vehicle alone, added dropwise onto the tumors on days 1, 2, 4, 5, and 7, and co‐treated with mis‐siRNAs or ATG16‐L1 siEx8 siRNAs on days 3 and 6. The concentration of gefitinib was defined based on the results of a pilot study in which we treated engrafted sensitive PC9 and resistant PC9 GR4 cells with vehicle (control) or various concentrations of gefitinib every day for 9 days (20 eggs for each group). Tumors were weighed at the end of the treatment. We choose the concentration of gefitinib (150 μm) that induced a significant decrease in tumor weight in PC9 cells (45% of decrease in that case) and has no effect in PC9 GR4 cells. The dosing schedule of gefitinib was based on this study. On day 9 of the co‐treatment study, the upper portion of CAM was removed, and tumors were cut away from normal CAM tissue and weighed. Tumors were frozen at −80 °C for RNA extraction and RT‐PCR analyses. Of note, we did not observe evidence of toxicity (no deleterious effect of the siRNA on the embryo) and no malformation and/or element of potential toxicity (local inflammation at the treatment site, abnormal size of the embryo, etc, …,) during the macroscopic observation of the embryos after collection of the samples.

### 2.12 Statistical analysis

All data are the mean ± SD of at least three independent experiments. Statistical comparisons between two groups were conducted using a two‐tailed unpaired t test or Mann–Whitney test as indicated in the figure legends. Statistical significance was noted as *P < 0.05; **P < 0.01; ***P < 0.001; ****P < 0.0001. ns stands for not statistically significant. All analyses were performed using graphpad prism software (GraphPad Software Inc.).

## 3 Results

### 3.1 Cells with acquired resistance to EGFR‐TKI overexpress the ATG16‐L1 β splicing variant

**Fig. 1:**
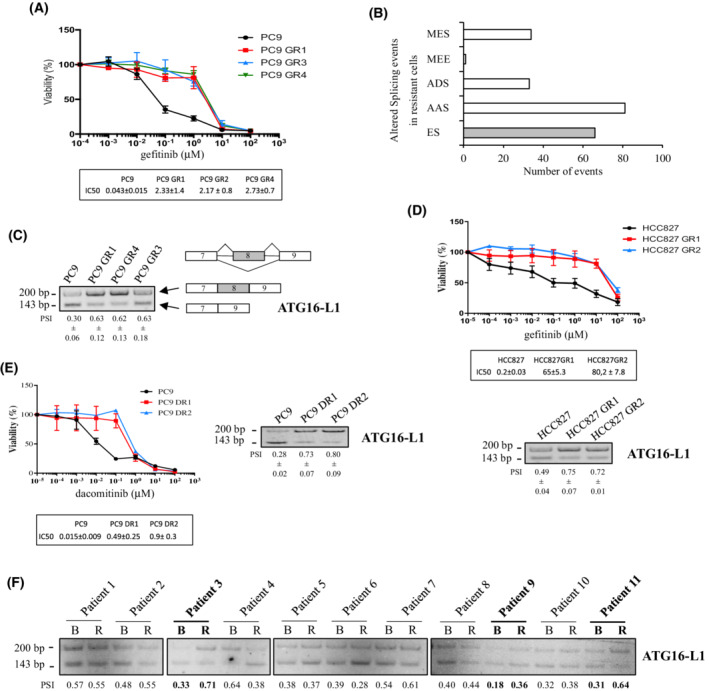
A splicing variant of ATG16‐L1 that retains exon 8 accumulates in EGFR‐TKI–resistant cells. (A) MTS cell viability assays showing resistance of PC9 GR cells compared with PC9 cells. IC50 *n* = 3. Mean ± SD. (B) RNA sequencing analysis reveals differential splicing events in resistant clones (PC9 GR1/3/4 used as simplicate) as compared to the original TKI‐sensitive cells (PC9 in triplicate). Alternative splicing (AS) events were categorized according to the AS type (MES, multiexon skipping; MEE, mutually exclusive exons; ADS, alternative donor site; AAS, alternative acceptor site; ES, exon skipping). (C) RT‐PCR analysis showing accumulation of ATG16‐L1 splicing variant that retains exon 8 in resistant clones (PC9 GR1/3/4) as compared to parental sensitive cells (PC9). Percent spliced‐in (PSI) is indicated below the PCR blots. *N* = 3. Mean ± SD. (D, E) MTS survival assay (upper panel) and RT‐PCR analysis of the ATG16‐L1 splicing switch (lower panel) in (D) gefitinib‐resistant HCC827‐derived clones (HCC827 GR1 and GR2) and (E) dacomitinib‐resistant PC9‐derived clones (PC9 DR1 and DR2). IC50 *n* = 3. Mean ± SD. (F), RT‐PCR analysis of the ATG16‐L1 splicing switch in paired tumor samples from 11 NSCLC patients before EGFR‐TKI treatment (B) and at relapse (R). Percent spliced‐in (PSI) is indicated below the PCR blots (*n* = 1). Accumulation of ATG16‐L1 Ex8 is observed at relapse in patient tumor samples 3, 9 and 11 (bold). [Colour figure can be viewed at wileyonlinelibrary.com]

**Fig. 2:**
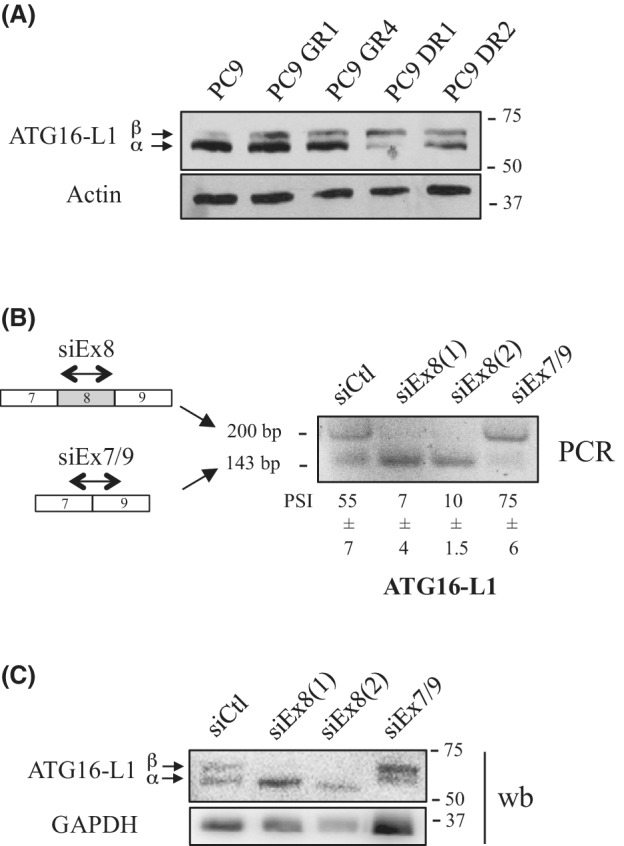
Altered splicing favoring inclusion of exon 8 in ATG16‐L1 mRNA leads to the accumulation of the ATG16‐L1 β protein isoform in resistant cells. (A) Indicated cell lines were subjected to immunoblotting using ATG16‐L1 antibody. Actin was used as a loading control (*n* = 3). (B, C) PC9 GR1 cells were transfected for 72 h with a control siRNA (siCtl) or two different siRNAs targeting exon 8 [siEx8(1) and siEx8(2)], or with a siRNA targeting the exon 7/9 junction (siEx7/9) of ATG16‐L1. Of note, a second siEx7/9 could not be designed because of the junction sequence. (B) RT‐PCR was performed on total RNA extracts. Percent spliced‐in (PSI) is indicated below the PCR blots. *N* = 3. Mean ± SD. (C) Expression of ATG16‐L1 was analyzed by western blotting, using GAPDH as a loading control (*n* = 3).

To address the role of altered splicing in acquired resistance to EGFR‐TKI, we generated a mass culture of PC9 cells resistant to EGFR inhibition using gefitinib as previously described [39] and then isolated resistant clones (PC9 GR) (Fig. 1A). We focused our study on three PC9 GR clones (PC9 GR1/GR3/GR4) that did not carry the T790M mutation nor other common mechanisms of acquired resistance (see Materials and methods section). High‐throughput RNA sequencing (RNA‐seq) was performed on sensitive PC9 cells (in triplicate) and resistant PC9 GR cells using the GR1, GR3, and GR4 clones as simplicate. Differential expression of splicing variants (defined as significantly altered splicing events in PC9 GR cells as compared to PC9 cells) was retrieved from the RNA‐seq datasets and categorized into five classes as previously described [41]: exon skipping (ES), alternative acceptor site (AAS), alternative donor site (ADS), mutually exclusive exons (MEE) and multi‐exon skipping (MES). We found a total of 212 differential splicing events, comprising 80 AAS, 65 ES, 33 MES, 33 ADS, and 1 MEE events (Fig. 1B and Table S3). Exon skipping is one of the most frequent alternative splicing events detected in cancer [17]. As it was also frequently observed in our resistant clones (Fig. 1B), we decided to further focus on this splicing event. Using RT‐PCR and primers surrounding the skipped exons, we validated 44 of the 54 ES tested events, revealing a higher rate of inclusion or exclusion of exons in PC9 GR cells as compared to PC9 cells (Fig. S1). Among genes affected by ES, we identified the autophagy‐related 16‐like 1 (ATG16‐L1) gene as a potent target of interest. Indeed, ATG16‐L1 is a critical regulator of autophagy, and the role of autophagy in response to EGFR‐TKI treatment remains a subject of debate in NSCLC with either cytotoxic or cytoprotective properties [34, 35, 36, 37, 38, 44]. In PC9 GR cells, increased expression of a splicing variant of ATG16‐L1 retaining exon 8 (referred to herein as ATG16‐L1 Ex8) was observed as compared to PC9 cells (Fig. 1C). Overexpression of ATG16‐L1 Ex8 was also found in gefitinib‐resistant clones generated from HCC827 (HCC827 GR1/GR2), another lung cancer cell line expressing the EGFR‐TKI–sensitizing Del19 mutation (Fig. 1D), and in PC9‐derived clones that had been rendered resistant to the 2^nd^ generation EGFR‐TKI Dacomitinib (PC9 DR1/DR2) (Fig. 1E). To go further, we took advantage of a small cohort of 11 NSCLC patients who had received EGFR‐TKI, and for which paired tumor tissue before treatment and at relapse were available. Heterogeneous levels of ATG16‐L1 Ex8 mRNA were found across tumor samples before treatment. Strikingly, however, we observed overexpression of ATG16‐L1 Ex8 in the tumors of 3/11 patients at relapse following EGFR‐TKI treatment compared with paired tumor samples before treatment (Fig. 1F). Moreover, when using an antibody that recognizes an epitope in the N‐terminal sequence of ATG16‐L1, immunoblotting revealed two protein bands with very closed molecular weights in our tumor cell lines (Fig. 2A). Interestingly, the resistant PC9 GR1 and PC9 GR4 clones expressed higher levels of the slower‐migrating band as compared to the parental (nonresistant) PC9 cells, and similar results were observed in PC9 DR1/DR2 clones (Fig. 2A). To test whether these protein isoforms were translated from ATG16‐L1 isoforms that retain or not the exon 8, we transfected PC9 GR1 clones with different siRNAs specifically targeting either exon 8 [siEx8(1) and siEx8(2)] or the exon 7/9 junction (siEx7/9). After knockdown of ATG16‐L1 Ex8 as verified by RT‐PCR (Fig. 2B), the slower‐migrating band in immunoblots was strongly decreased (Fig. 2C). In parallel, the siRNA targeting the exon7/9 junction diminished the expression level of the faster‐migrating band (Fig. 2B,C). Of note, neutralization of either ATG16‐L1 isoforms (*e.g*., containing or not exon 8) induced the accumulation of the other one, at both the mRNA and protein levels, suggesting a feedback control between isoforms. According to Ensembl data base, our PCR and western blot results strongly suggested that resistant cell lines overexpress the ATG16‐L1‐203 transcript also called β‐isoform that retains exon 8 (residues 266–284), as opposed to the α‐isoform (ATG16‐L1‐205 transcript), which lacks exon 8. Because exon 8 contains only 18 residues, the α‐ and β‐isoforms code for proteins with very similar molecular weight (around 66 KDa and 68 KDa, respectively). As a whole, our results showed that lung tumor cells with acquired resistance to EGFR‐TKI accumulate the ATG16‐L1 β‐isoform.

### 3.2 Inhibition of ATG16‐L1 β induces apoptosis in response to EGFR‐TKI and limits tumor growth

**Fig. 3:**
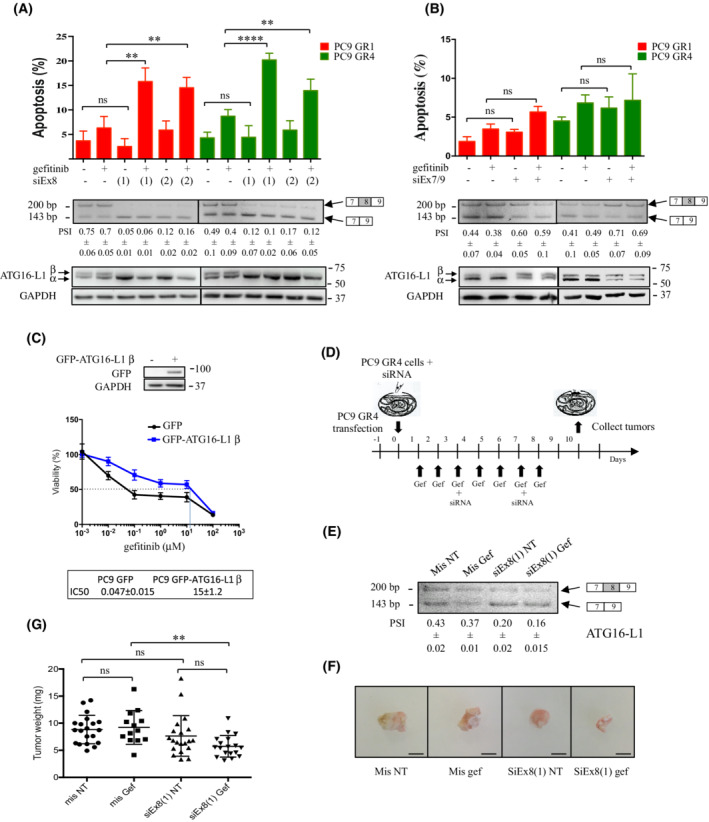
Neutralization of ATG16‐L1 β restores apoptosis and inhibits the growth of tumor xenografts in response to gefitinib. (A, B) PC9 GR1 and GR4 cells were transfected with control (mis‐siRNA) or specific siRNAs against (A) exon 8 [siEx8 (1), siEx8 (2), (1), and (2) represent two different siRNAs] or (B) the exon7/exon9 junction (siEx7/9) of ATG16‐L1, and grown with or without gefitinib (0.1 μm) for 72 h, as indicated. Levels of apoptosis were determined after cleaved caspase‐3 staining and FACS analysis (upper panels) (*n* = 5). Data, mean ± SD per treatment condition; unpaired t test; ****P ≤ 0.0001; **P ≤ 0.01; ns, not significant. Representative RT‐PCR and Western blots for the validation of ATG16‐L1 α and β neutralization are shown (lower panels). Percent spliced‐in (PSI) is indicated below the PCR blots. *N* = 3. Mean ± SD. GAPDH was used as a loading control. (C) PC9/GFP and PC9/GFP‐ATG16‐L1 β cells were treated with gefitinib and cell viability was studied using MTS assays. IC50 *n* = 3. Mean ± SD. Western blotting was performed to measure GFP‐ATG16‐L1 β overexpression. (D) Schematic representation of the strategy used for the *in ovo* engraftment and treatment of PC9 GR4 clones into chicken eggs. (E) Representative RT‐PCR for the validation of ATG16‐L1 β neutralization. Percent spliced‐in (PSI), mean ± SD per treatment condition (*n* = 3). (F) Representative images of tumor xenografts at the end of treatment. Scale bar: 2 mm. (G) Tumor weight for each treatment condition. Data, mean ± SD per treatment condition; Mann–Whitney test; **P ≤ 0.01; ns, not significant. [Colour figure can be viewed at wileyonlinelibrary.com]

To assess whether the high expression level of ATG16‐L1 β was involved in acquired resistance to EGFR‐TKI, we first transfected PC9 GR1 and GR4 cells with siEx8 (or control mis‐siRNA) and analyzed apoptosis using active caspase 3 staining and FACS analysis in cells grown with or without gefitinib. Transfection with siEx8 alone did not modify apoptotic levels. However, when cells were co‐cultured with gefitinib, apoptosis occurred (Fig. 3A). Induction of apoptosis was confirmed using annexin V staining and PARP immunoblotting (Fig. S2). The same results were obtained in PC9 DR2 cells treated or not with dacomitinib (Fig. S3). In contrast, transfecting PC9 GR1 or GR4 cells with siEx7/9 had no effect whatever the conditions (Fig. 3B). To go further, we established PC9 cells stably overexpressing green fluorescent protein (GFP)‐tagged human ATG16‐L1 β. Using survival assays, we showed that expression of GFP‐ATG16‐L1 β protected PC9 cells from gefitinib (Fig. 3C) in agreement with a role of ATG16‐L1 β overexpression in resistance to EGFR‐TKI. To validate these results *in vivo*, we engrafted PC9 GR4 cells onto chicken chorioallantoid membranes (CAM). This fast, sensitive, and reproducible *in vivo* system has been shown to mimic results obtained in mice [45, 46]. At 24 h prior to engrafting, cells were transfected with mis‐siRNA or siEx8. Once engrafted, tumors were treated every day over 9 days with vehicle (control) or gefitinib, and received mis‐siRNA or siEx8 siRNA twice during the treatment (Fig. 3D). Tumors recovered from the upper CAMs were weighted and analyzed by RT‐PCR to validate the neutralization of ATG6‐L1 β (Fig. 3E). Gefitinib treatment or siEx8 transfection alone had no significant effects on tumor growth. In contrast, co‐treating tumors with siEx8 and gefitinib significantly reduced tumor weight (Fig. 3F,G). As a whole, these results showed that accumulation of ATG6‐L1 β inhibits gefitinib‐induced apoptosis in cells with acquired resistance to EGFR‐TKI and promotes tumor growth.

### 3.3 Resistant clones display reduced basal autophagy

**Fig. 4:**
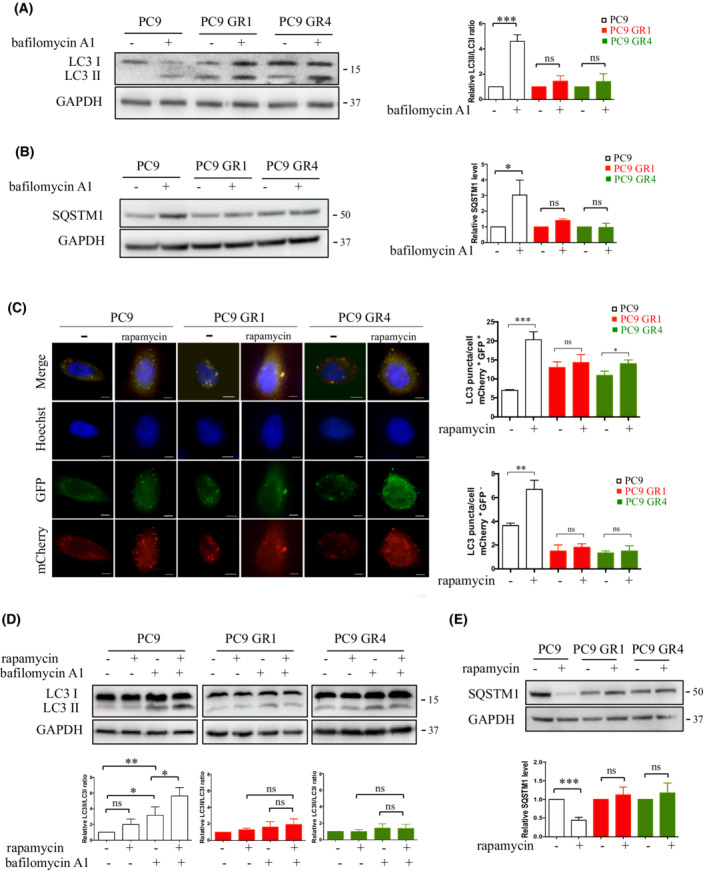
Resistant clones have a reduced autophagy flux. (A, B) Cells were grown with or without bafilomycin A1 (0.1 μm) for 2 h (A) or 4 h (B). Representative western blots of LC3 and SQSTM1 expression are shown. GAPDH was used as a loading control. LC3‐II/LC3‐I and SQSTM1/GAPDH ratios were calculated in each condition using imagej. Relative LC3‐II/LC3‐I and SQSTM1/GAPDH ratios are represented in comparison with the untreated condition in each cell line, which was arbitrarily assigned to 1 (*n* = 3). Data, mean ± SD per treatment condition; unpaired *t* test; *P ≤ 0.05; ***P ≤ 0.001; ns, not significant. (C) Representative images of PC9 and PC9 GR cells transfected with mCherry‐GFP‐LC3 and treated with or without (−) rapamycin (1 μm) for 2 h. scale bar: 10 μm. Quantitative analysis of the number of GFP^+^mCherry^+^ (yellow) puncta and GFP^−^mCherry^+^ (red) puncta per cell. Histograms represent the mean ± SD of 3 independent experiments. Unpaired *t* test; *P < 0.05, **P < 0.01, ***P < 0.001; ns, not significant. (D, E) Cells were grown with or without bafilomycin A1 (0.1 μm) and/or rapamycin (1 μm) for *2* h. Representative western blots of LC3 and SQSTM1 expression are shown. GAPDH was used as a loading control. LC3‐II/LC3‐I and SQSTM1/GAPDH ratios were calculated in each condition using imagej. Relative LC3‐II/LC3‐I and SQSTM1/GAPDH ratios are represented in comparison with the untreated condition in each cell line, which was arbitrarily assigned to 1 (*n* = 3). Data, mean ± SD per treatment condition; unpaired t test; *P ≤ 0.05; **P ≤ 0.01; ***P ≤ 0.001; ns, not significant. [Colour figure can be viewed at wileyonlinelibrary.com]

As ATG16‐L1 is an essential component of autophagosome formation [25], we wondered whether autophagy may be deregulated in resistant cells. First, we measured autophagic activity in sensitive and resistant cells. The mammalian autophagy protein, LC3, is a marker of autophagosomes. Upon induction of autophagy, endogenous LC3‐I is converted into a lipidated LC3‐II form, which is recruited and integrated into the membranes of the autophagosome [47]. Western blotting of LC3 and determination of a, LC3‐II/LC3‐I ratio is usually considered an accurate measure of autophagic flux. The relevant parameter in LC3 assays is the difference in the amount of LC3‐II in the presence or absence of saturating levels of autophagic inhibitors such as bafilomycin A1, which can be used to examine the transit of LC3‐II through the autophagic pathways: if flux is occurring, the amount of LC3‐II (and as a consequence the LC3‐II/LC3‐I ratio) will be higher in the presence of the inhibitor. SQSTM1/p62 is also used as a protein marker for the study of autophagy. The SQSTM1 protein serves as a link between LC3 and autophagic substrates and becomes incorporated into the completed autophagosome and degraded in autolysosomes [48]. Therefore, decreased levels of SQSTM1 correlate with autophagic flux whereas in the presence of bafilomycin A1 the levels will be higher. When PC9 cells were treated with bafilomycin A1, the LC3‐II/LC3‐I ratio was higher as compared to untreated cells (Fig. 4A) and an enhancement of SQSTM1 protein level was observed (Fig. 4B). These results indicated that autophagic carrier flux was occurring. In contrast, bafilomycin A1 did not significantly modify the LC3‐II/LC3‐I ratio (Fig. 4A) nor the SQSTM1 protein level (Fig. 4B) in PC9 GR cells, suggesting that PC9 GR cells have a reduced autophagic activity compared with PC9 cells. We further studied the relative levels of autophagic flux in PC9 and PC9 GR cells using transient transfection of the mCherry‐GFP‐LC3 vector and treatment with the autophagic activator rapamycin. The association of lipidated LC3‐II with autophagosomal membranes results in the formation of punctate organelles that can be quantified by fluorescence microscopy [49]. Autophagosomes are visualized by yellow puncta (mCherry ^+^ GFP ^+^) and autolysosomes are visualized by red puncta (mCherry ^+^ GFP ^−^) as the GFP signal is quenched in the acidic environment of lysosomes. Upon rapamycin treatment, PC9 cells displayed an increased number of yellow and red puncta compared with untreated conditions (Fig. 4C), revealing increased autophagic flux. In contrast, the number of yellow and red puncta per cell remained approximately the same in PC9 GR cells cultured with or without rapamycin. Consistently, immunoblotting data showed an enhanced LC3‐I to LC3‐II conversion (Fig. 4D) and a decreased SQSTM1 expression level (Fig. 4E) in PC9‐sensitive cells treated with rapamycin but not in resistant PC9 GR cells. As a whole, these results indicated that basal autophagic flux levels are reduced in cells with acquired resistance to EGFR‐TKI compared with sensitive parental cells.

### 3.4 Gefitinib‐induced autophagy is decreased in resistant cells independently of mTOR

**Fig. 5:**
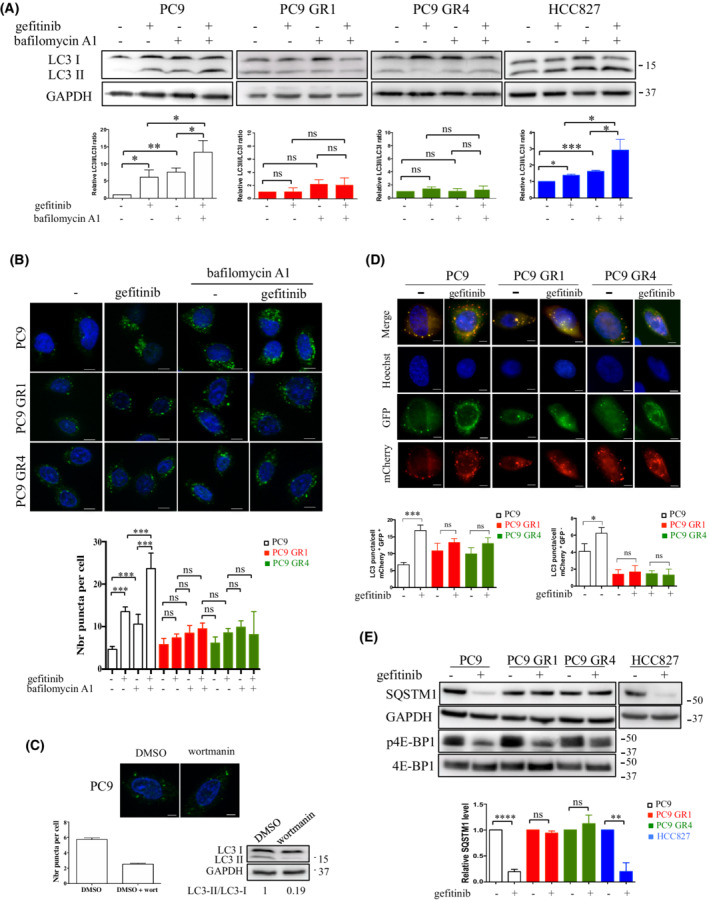
Induction of autophagy by EGFR‐TKI is impaired in resistant clones. (A, B, E) Cells were cultured with (+) or without (−) gefitinib (0.1 or 0.5 μm) and/or bafilomycin A1 (0.1 μm) for 2 h. (A) Representative western blots of LC3 are shown. GAPDH was used as a loading control. LC3‐II/LC3‐I ratios were calculated in each condition. Relative LC3‐II/LC3‐I ratios are represented in each cell line in comparison with the untreated condition, which was arbitrarily assigned to 1. Data, mean ± SD per treatment condition (*n* = 3) unpaired *t* test; *P ≤ 0.05; **P ≤ 0.01; ***P ≤ 0.001; ns, not significant. (B) Representative images of LC3 puncta in indicated cell lines after immunofluorescent staining with anti‐LC3 antibody (green). Nuclei were stained with Hoechst (blue). Scale bar: 10 μm. Quantification of LC3 puncta in each condition (≥ 50 cells analyzed per sample, *n* = 3) is shown. Data, mean ± SD per treatment condition; unpaired t test; ***P ≤ 0.001; ns, not significant. (C) PC9 cells were grown with HBSS ± wortmannin (0.5 μm) for 1 h. Representative images of LC3 puncta following immunofluorescent staining with anti‐LC3 antibody (green). Nuclei were stained with Hoechst (blue). Scale bar: 10 μm. Quantification of LC3 puncta in each condition (≥ 50 cells analyzed per sample, *n* = 3) is shown. Data, mean ± SD per treatment condition. Inhibition of LC3‐II expression by wortmannin was studied by western blotting. (D) Representative images of PC9 and PC9 GR cells transfected with mCherry‐GFP‐LC3 and treated with or without (−) gefitinib (0.1 μm) for 2 h. Scale bar: 10 μm. Quantitative analysis of the number of GFP^+^mCherry^+^ (yellow) puncta and GFP^−^mCherry^+^ (red) puncta per cell. Histograms represent the mean ± SD of 3 independent experiments. Unpaired *t* test. *P < 0.05; ***P < 0.001. ns, not significant. (E) Representative western blots of SQSTM1, 4E‐BP1, and phospho‐4E‐BP1 are shown. GAPDH was used as a loading control. SQSTM1/GAPDH ratios were calculated in each condition. Relative SQSTM1/GAPDH ratios are represented in comparison with the untreated condition in each cell line, which was arbitrarily assigned to 1 (*n* = 3). Data, mean ± SD per treatment condition; unpaired t test; **P ≤ 0.01; ****P ≤ 0.0001; ns, not significant. [Colour figure can be viewed at wileyonlinelibrary.com]

To further deepen the role of autophagy in resistance to EGFR‐TKI, we studied autophagic activity following gefitinib treatment. In PC9‐sensitive cells, gefitinib increased the LC3‐II/LC3‐I ratio (Fig. 5A). A higher increase was observed with treatment plus bafilomycin A1 suggesting that gefitinib enhances autophagic flux. In addition, the LC3‐II/LC3‐I ratio was higher in cells treated with gefitinib plus bafilomycin A1 compared with cells treated with bafilomycin A1 alone, indicating that gefitinib may also increase the synthesis of autophagy‐related membranes. Same results were obtained in HCC827, another lung cancer cell line that carries a EGFR‐TKI–sensitizing mutation. In contrast, the LC3‐II/LC3‐I ratio was not modified after treatment of the PC9 GR cells with either drug alone or with the co‐treatment. Same results were obtained using indirect immunofluorescent staining of endogenous LC3 in which the formation of autophagosomes was monitored through the appearance of LC3 puncta. Gefitinib increased the number of LC3 puncta in sensitive PC9 cells but not in resistant PC9 GR cells (Fig. 5B). Treating PC9 cells with the autophagic inhibitor wortmannin reduced the number of LC3 puncta (Fig. 5C), suggesting that the LC3‐positive structures are autophagic vesicles. Measurement of autophagic flux was also performed using mCherry‐GFP‐LC3 transfection experiments. PC9 cells transfected with mCherry‐GFP‐LC3 displayed an increased number of yellow and red puncta per cell when treated with gefitinib compared with untreated conditions (Fig. 5D). This indicated that gefitinib stimulated autophagosome formation and their maturation into autolysosomes. In contrast, the number of yellow and red puncta per cell stayed pretty much the same in gefitinib‐treated PC9 GR cells compared with untreated conditions. Immunoblotting of SQSTM1 confirmed that autophagy activation was impaired in gefitinib‐treated PC9 GR cells compared with PC9 cells (Fig. 5E). Because the mTOR inhibitor rapamycin induces autophagy in parental PC9 but not in PC9 GR cells (Fig. 4C–E), we asked whether signaling in PC9 GR cells could result in lower dependency on mTOR compared with parental cells. Therefore, we compared the effects of gefitinib on mTOR activity as measured by phosphorylation of the mTOR substrate, 4E‐BP1. We showed that gefitinib induces dephosphorylation of 4E‐BP1 in both PC9 and PC9 GR cells (Fig. 5E). These results suggested that gefitinib‐induced autophagy is impaired in TKI‐resistant cells in a manner that is independent of mTOR.

### 3.5 Inhibition of ATG16‐L1 β restores gefitinib‐induced autophagy in resistant cells

**Fig. 6:**
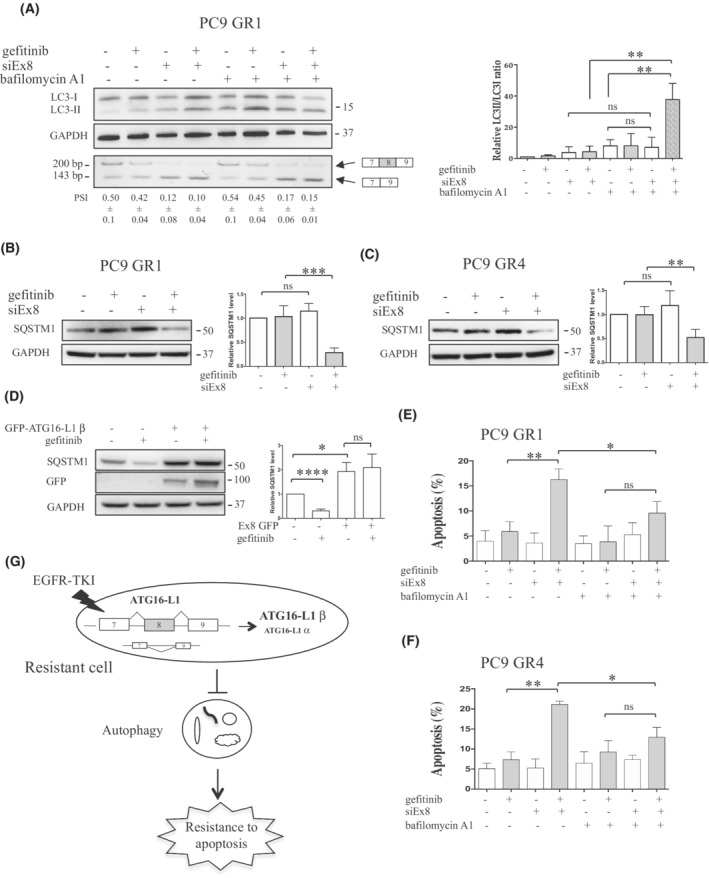
Neutralization of ATG16‐L1 β restores autophagy activation and apoptosis in response to EGFR‐TKI. (A–C) Cells were transfected with control (−, mis‐siRNA) or specific siRNA against exon 8 (siEx8) of ATG16‐L1 β. Seventy‐two hours post‐transfection growth medium was changed, and cells were cultured with (+) or without (−) gefitinib (0.1 μm) and bafilomycin A1 (0.1 μm) for an additional 2 h (A) or 4 h (B, C). Representative RT‐PCR for ATG16‐L1 β neutralization are illustrated. Percent spliced‐in (PSI), mean ± SD per treatment condition (*n* = 3) (A, lower panel). Representative western blots of LC3 and SQSTM1 expression are shown. GAPDH was used as a loading control. LC3‐II/LC3‐I and SQSTM1/GAPDH ratios were calculated in each condition using imagej. Relative LC3‐II/LC3‐I and SQSTM1/GAPDH ratios are represented in each cell line in comparison with the untreated condition, which was arbitrarily assigned to 1 (*n* = 4). (D) PC9/GFP and PC9/GFP‐ATG16‐L1 β cells were treated with gefitinib (0.1 μm) for 24 h. Autophagy was studied using SQSTM1 immunoblotting and quantified as described in (B, C). GFP‐ATG16‐L1 β expression was measured using GFP antibody. (E, F) PC9 GR1/GR4 cells were transfected with control (−, mis‐siRNA) or specific siRNA against exon 8 (siEx8) of ATG16‐L1 β and grown with (+) or without (−) gefitinib (0.1 μm) and bafilomycin A1 (5 nm) for 72 h. Apoptosis was studied after cleaved caspase 3 staining and FACS analysis (*n* = 4). (A–F) Data, mean ± SD per treatment condition; unpaired t test; *P ≤ 0.05; **P ≤ 0.01; ***P ≤ 0.001; ****P ≤ 0.0001. ns, not significant. (G) A working model of acquired resistance to EGFR‐TKI. Chronic exposure of lung tumors to EGFR‐TKI induces deregulated splicing of ATG16‐L1 in favor of the β isoform that retains exon 8. This blocks the induction of autophagy in response to EGFR‐TKIs and inhibits apoptosis, thereby inducing TKI resistance.

Finally, we tested whether overexpression of ATG16‐L1 β in resistant cells was involved in the inhibition of gefitinib‐induced autophagy. PC9 GR1 cells were transfected with siEx8 and autophagy assays were performed with and without gefitinib. Transfection with siEx8 did not significantly modify the LC3‐II/LC3‐I ratio either with or without bafilomycin A1 (Fig. 6A). However, when gefitinib was added (siEx8/gefitinib), a strong enhancement of the LC3‐II/LC3‐I ratio was observed with co‐treatment with bafilomycin A1 compared with siEx8/gefitinib alone and to bafilomycin A1 alone. In addition, SQSTM1 protein levels were decreased in siEx8/gefitinib PC9 GR1 cells compared with siEx8 PC9 GR1 cells (Fig. 6B), and in the PC9 GR4 model (Fig. 6C). Therefore, neutralization of ATG16‐L1 β restored autophagy induction in response to gefitinib in resistant cells. In agreement with these results, gefitinib‐induced autophagy was not observed in parental PC9 cells that overexpressed GFP‐ATG16‐L1 β (Fig. 6D). Importantly, pharmacological inhibition of autophagy using bafilomycin A1 partially prevented the re‐sensitization to apoptosis in both siEx8/gefitinib PC9 GR1 and PC9 GR4 cells (Fig. 6E and F). These results suggested that altered splicing of ATG16‐L1 in favor of the expression of ATG16‐L1 β blocks the induction of autophagy in response to EGFR‐TKI, and as a consequence inhibits apoptosis thereby promoting resistance (Fig. 6G).

## 4 Discussion

The remarkable success of EGFR‐TKIs for the treatment of lung adenocarcinoma patients with advanced‐mutated EGFR has been undermined by the invariable development of acquired resistance to these drugs. Thus, there is a need to address the multi‐factorial biological bases of resistance in order to design tailored treatments to forestall tumor evolution and to maximize survival. In recent years, there has been mounting evidence that alternative splicing plays a role in the cancer drug response. In melanoma, splice variants of BRAF lacking the RAS‐binding domain confer resistance to vemurafenib [50]. In NSCLC, a splicing isoform of BIM that lacks the BH3 domain was recently associated with intrinsic resistance to gefitinib in NSCLC [51]. In this study, we showed that NSCLC cells with acquired resistance to EGFR‐TKIs display aberrant splicing patterns as compared to original sensitive cells. More importantly, we found that altered exon skipping events are commonly observed in resistant clones thereby identifying new potential mechanisms of resistance. Notably, we found accumulation of the ATG16‐L1 β splicing isoform in resistant cells and showed that its downregulation restored sensitivity to EGFR‐TKI by inducing apoptosis. Importantly, overexpression of this variant was observed in lung tumors from 3 of 11 NSCLC patients at relapse following EGFR‐TKI treatment. These results suggest that splicing regulation of *ATG16‐L1* is a therapeutic vulnerability for lung tumors with acquired resistance to EGFR‐TKI.

Compiling data from different databases highlight the existence of several ATG16‐L1 splicing isoforms in humans, some of them contain exon 8 and encode different protein isoforms. According to this, our results suggested that NSCLC co‐express the α‐ and β‐ isoforms of ATG16‐L1 that differ by the retention of exon 8 in the β‐ isoform only, and that cells with acquired resistance to EGFR‐TKI overexpress the ATG16‐L1 β‐isoform. We noticed that siRNA targeting ATG16‐L1 β (siEx8) provokes an increase in the expression of ATG16‐L1 α. Therefore, the increase of the α‐isoform of ATG16‐L1 could explain the biological effects of the siEx8. However, in tumor patients with an increased ATG16‐L1 β/α ratio at relapse, overexpression of the β‐isoform is not associated with a decrease of the α isoform (Fig. 1F). In addition, overexpressing ATG16‐L1 α in PC9‐sensitive cells did not enhance their sensitivity to EGFR‐TKI (data not shown). Therefore, we consider that the decrease of ATG16‐L1 β rather than the increase of ATG16‐L1 α is mainly responsible for the effects of siEx8 in resensitizing resistant cells to EGFR‐TKI.

ATG16‐L1 is critical for LC3 lipidation and autophagosome biogenesis. Using regulators of autophagy (bafilomycin A1, rapamycin), we showed that basal autophagic flux levels are reduced in resistant cells compared with sensitive ones. More importantly, we further found that gefitinib‐induced autophagy is impaired in resistant cells that accumulate ATG16‐L1 β, and showed that neutralization of ATG16‐L1 β restores autophagy induction in response to gefitinib. In addition, overexpression of ATG16‐L1 β in parental sensitive cells prevented gefitinib‐induced autophagy. A previous study demonstrated that correct stoichiometry of ATG16‐L1 complex components is critical for autophagosome biogenesis, and that overexpression of ATG16‐L1 β causes inhibition of autophagosome formation by a mechanism that remains to be determined [52]. We hypothesize that overexpression of ATG16‐L1 β in our resistant cells dysregulates autophagosome biogenesis in response to gefitinib thereby preventing autophagy induction. Further studies would be required to characterize the mechanism underlying the inhibitory effect of overexpressed ATG16‐L1 β on gefitinib‐induced autophagy.

Autophagy has been shown to be activated in response to EGFR‐TKI. However, the consequence of the induction of autophagy remains a subject of debate. Some studies suggested that autophagy induction enhances the response [34, 44], while others claimed that it restrains the growth inhibitory effect of EGFR‐TKIs [35, 36, 37, 38]. Strikingly, a great number of these studies were done in NSCLC cell lines that did not carry EGFR‐TKI–sensitive mutations (thus making the outcomes less relevant), and the role of autophagy in acquired resistance to first‐line EGFR‐TKI treatment was poorly addressed in TKI‐sensitive cells. Nihira et al. [53] previously reported that activation of autophagy (through LC3A upregulation) contributes to acquired resistance to erlotinib. Conversely, our data showed that autophagy may be impaired in NSCLC cells with acquired resistance to EGFR‐TKI. In our study, impaired autophagy was associated with increased expression of ATG16‐L1 β. Downregulation of ATG16‐L1 β restored autophagy activation and sensitivity to EGFR‐TKI treatment by inducing apoptosis. Apoptosis was partially prevented when autophagy was inhibited by treating ATG16‐L1 β‐depleted cells with Bafilomycin implying that autophagy is contributing to cell death. Moreover, in parental sensitive cells, EGFR‐TKI resistance was conferred by overexpression of ATG16‐L1 β that blocked autophagy activation following TKI treatment. Together, our results suggest that the induction of autophagy may contribute to EGFR‐TKI‐induced cell death in sensitive NSCLC cell lines, and that suppression of autophagy (or the inability to increase autophagic flux above basal levels) promotes the development of acquired resistance to EGFR‐TKI. These findings are supported by a study from Yongjie Wei et al. [44] who showed that autophagy inhibition, through constitutive expression of a beclin 1 phosphomimetic mutant, increases clonogenic survival and TKI resistance in sensitive NSCLC cell lines and xenografts. They are also in agreement with Fung C et al.'s study [31], which reported that autophagy is not robustly activated in cells highly resistant to EGFR‐TKI, and that co‐treatment with autophagy inducers can partially restore sensitivity to EGFR‐TKI.

## 5 Conclusions

Our study shows that splicing deregulation of ATG16‐L1 leads to acquired resistance of NSCLC cells to EGFR‐targeted therapies (see Fig. 6F). This may block the induction of autophagy and inhibit apoptosis in response to EGFR‐TKIs, thereby promoting cell survival and tumor growth. These findings could explain the conflicting results of clinical trials using autophagy inhibitors, and indicate that strategies to correct ATG16‐L1 splicing and/or increase autophagy in combination with EGFR‐TKI may represent new therapeutic approaches to overcome resistance in selected NSCLC patients.

## Conflict of interest

Dr Toffart reports grants, personal fees, and nonfinancial support from Roche and Pfizer and personal fees and nonfinancial support from Boehringer Ingelheim and Astra Zeneca during the conduct of the study; and personal fees and nonfinancial support from Novartis, Vifor Pharma and MSD, grants and personal fees from BMS, outside the submitted work. The sponsors had no role in the design, execution, interpretation, or writing of the study.

## Author contributions

SG, BE, and DA involved in conception and design; ASH, AP, PP, LL, FDF, MGL, ACT, AR, and CB involved in data acquisition; ASH, AP, PP, LL, AR, CB, DA, BE, and SG involved in data analysis and interpretation; SG, BE, and DA involved in writing and/or revision of the manuscript; SG and BE involved in funding acquisition; SG involved in study supervision.

6

### Peer Review

The peer review history for this article is available at https://publons.com/publon/10.1002/1878-0261.13229

## Supporting information


**Fig. S1.** Validation of RNA‐seq data.

**Fig. S2.** Neutralization of ATG16‐L1 β increases annexin V staining and induces PARP cleavage in response to gefitinib.

**Fig. S3.** Neutralization of ATG16‐L1 β restores apoptosis in response to dacomitinib.

**Table S1.** ATG16‐Ex8 status and clinical data of human samples.

**Table S2.** Genes for which altered exon skipping in PC9 GR‐resistant cells compared with PC9‐sensitive cells was validated by RT/PCR.

**Table S3.** Altered splicing events in PC9 GR‐resistant cells compared with PC9‐sensitive cells.

## Data Availability

All data generated or analyzed during this study are included in the published article and its supporting information files.
