# Correction to “Altered splicing of ATG16‐L1 mediates acquired resistance to tyrosine kinase inhibitors of EGFR by blocking autophagy in non‐small cell lung cancer”

**DOI:** 10.1002/1878-0261.70247

**Published:** 2026-03-27

In the manuscript by Hatat AS *et al*. [1], a horizontal flip of the ATG16‐L1 blot between Fig. 1C,D was identified in April 2023. Upon an independent investigation by the editorial office, the authors provided appropriate raw data and experimental repeats for both Fig. 1C,D. The authors acknowledged an oversight that occurred during the assembly of Fig. 1D, as the samples for the experiments in Fig. 1C,D were loaded onto the same agarose gel. Consequently, the authors have revised Fig. 1 to accurately represent the quantification shown in Fig. 1D. The corrected figure is amended below.

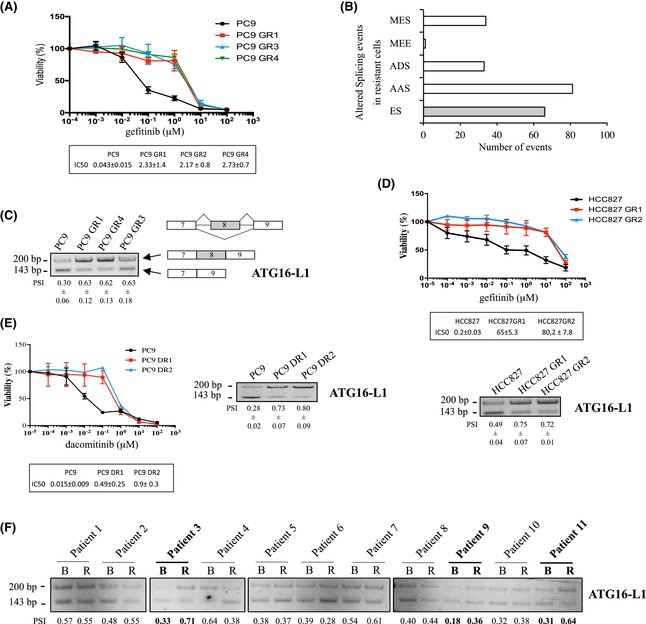

The correction of these errors has not altered the interpretation of the data and has not impacted the conclusions presented in the article. The authors agree to this corrigendum and apologize for any inconvenience caused.
