# Supplementary material for: Altered splicing of ATG16‐L1 mediates acquired resistance to tyrosine kinase inhibitors of EGFR by blocking autophagy in non‐small cell lung cancer
Source: Mol Oncol. 2022 Aug 30;16(19):3490–508. doi: 10.1002/1878-0261.13229 (PMC9533692; doi:10.1002/1878-0261.13229)
Supplement: Supplementary file 1 — Fig. S1. Validation of RNA‐seq data. [file MOL2-16-3490-s001.pdf]

Figure S2

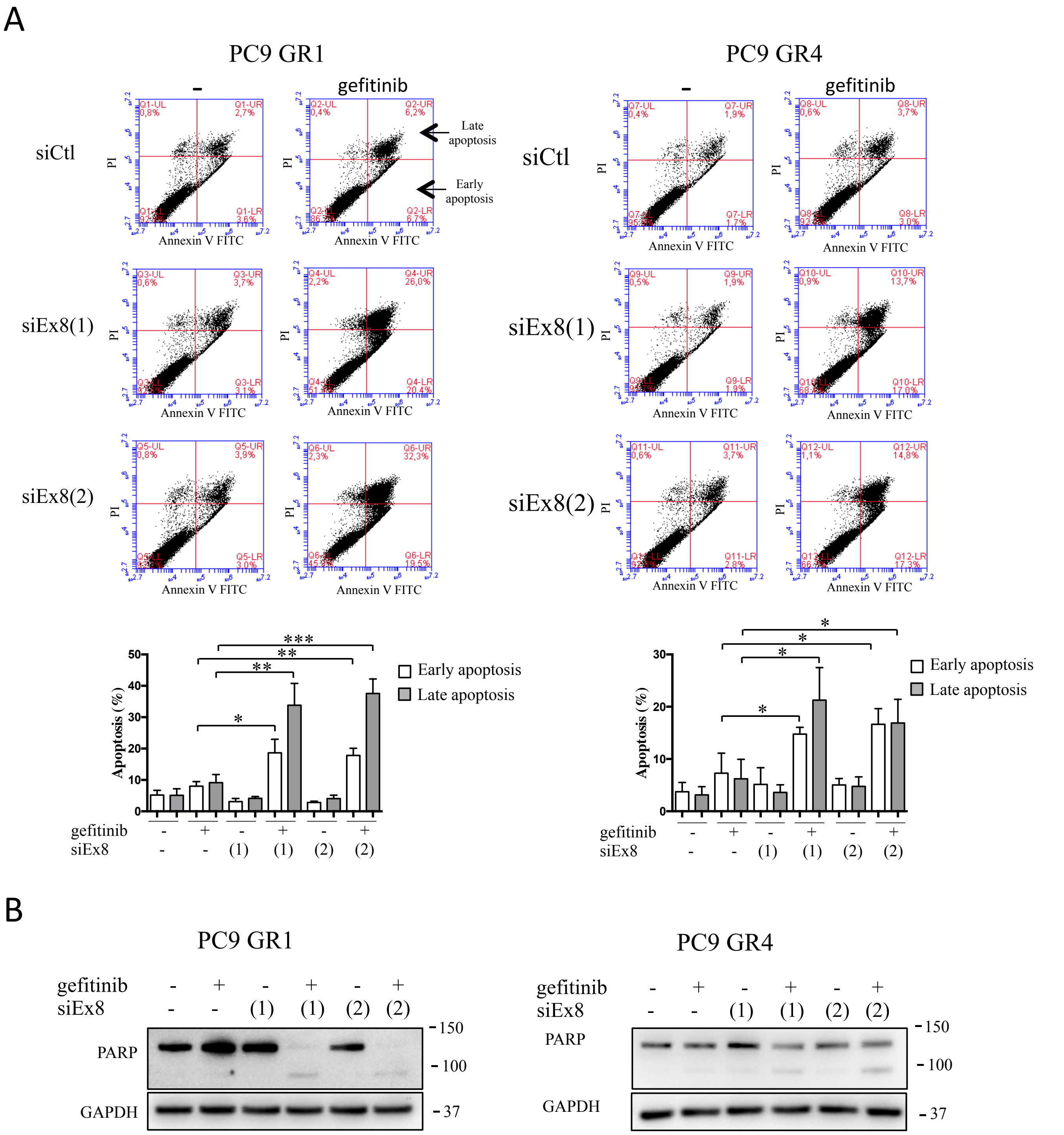

Neutralization of ATG16-L1 β increases annexin V staining and induces PARP cleavage in response to gefitinib

Resistant PC9 GR1 and GR4 cells were transfected with mismatch control (Ctl) siRNA or with siRNA targeting exon8 (siEx8 (1), siEx8 (2)) of ATG16-L1 and cultured for 72 hours with or without gefitinib (0.1µM). (A) Apoptosis was evaluated with the eBioscience[TM] Annexin V-FITC Apoptosis kit (In Vitrogen, Thermo Fischer Scientific) and analyzed by fluorescence-activated cell sorting (FACS) (n = 3). Data, mean ± SD per treatment condition, Unpaired t test, * $p \leq 0.05$ ** $p \leq 0.01$ *** $p \leq 0.001$ ns, non significant. (B) Representative western blots of PARP are shown. PARP cleavage indicates apoptosis. GAPDH was used as a loading control.
