# Supplementary material for: Altered splicing of ATG16‐L1 mediates acquired resistance to tyrosine kinase inhibitors of EGFR by blocking autophagy in non‐small cell lung cancer
Source: Mol Oncol. 2022 Aug 30;16(19):3490–508. doi: 10.1002/1878-0261.13229 (PMC9533692; doi:10.1002/1878-0261.13229)
Supplement: Supplementary file 2 — Fig. S2. Neutralization of ATG16‐L1 β increases annexin V staining and induces PARP cleavage in response to gefitinib. [file MOL2-16-3490-s006.pdf]

Figure S3

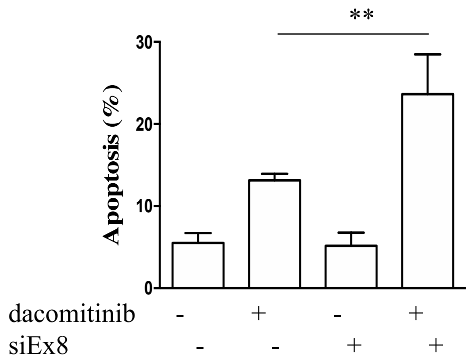

Neutralization of ATG16-L1 β restores apoptosis in response to dacomitinib
Resistant PC9 DR2 cells were transfected with mismatch control (-) siRNA or with siRNA targeting exon8 (siEx8) of ATG16-L1 and cultured for 72 hours with or without dacomitinib (0.01mM). Apoptosis was quantified after active caspase 3 staining and FACS analysis (upper panel) (n = 4). Data, mean ± SD per treatment condition, Unpaired t test,** $p \leq 0.01$. ns, non significant.
