# Supplementary material for: Altered splicing of ATG16‐L1 mediates acquired resistance to tyrosine kinase inhibitors of EGFR by blocking autophagy in non‐small cell lung cancer
Source: Mol Oncol. 2022 Aug 30;16(19):3490–508. doi: 10.1002/1878-0261.13229 (PMC9533692; doi:10.1002/1878-0261.13229)
Supplement: Supplementary file 3 — Fig. S3. Neutralization of ATG16‐L1 β restores apoptosis in response to dacomitinib. [file MOL2-16-3490-s004.pdf]

Table S1

Patients	EGFR exon 19 status	EGFR Exon 20	EGFR Exon 21	ATG16-L1 exon 8 retention	Treatment
1B	WT	WT	L858R*	-	
1R	WT	T790M*	L858R*	-	Tarceva
2B	E746_A750del*	WT	WT	-	
2R	E746_A750del*	WT	WT	-	Tarceva
3B	WT	WT	L858R*	-	
3R	WT	WT	L858R*	+	Iressa
4B	L747_S752 del*	WT	WT	-	
4R	L747_S752 del*	WT	WT	-	Iressa
5B	WT	WT	L858R*	-	
5R	WT	T790M*	L858R*	-	Tarceva
6B	E746_A750del*	WT	WT	-	
6R	E746_A750del*	T790M*	WT	-	Tarceva
7B	E746_A750del*	WT	WT	-	
7R	E746_A750del*	T790M*	WT	-	Iressa
8B	E746_S752delinsV*	WT	WT	-	
8R	E746_S752delinsV*	WT	WT	-	Giotrif
9B	E746_A750del*	WT	WT	-	
9R	E746_A750del*	T790M*	WT	+	Tarceva
10B	WT	WT	L858R*	-	
10R	WT	T790M*	L858R*	-	Tarceva
11B	WT	WT	L858R*	-	
11R	WT	WT	L858R*	+	Iressa

ATG16-Ex8 status and clinical data of human samples. ATG16-L1 splicing switch (exon 8 retention) was studied by RT/PCR in paired samples from eleven lung cancer patients before treatment (B) and at relapse (R). *HGVS nomenclature E746_A750del: c.2235_2249del; p.(Glu746_Ala750del) and c. 2236_2250del; p.(Glu746_Ala750del), L747_S752 del: c.2239_2256del; p.(Leu747_Ser752del), E746_S752delinsV: c.2237_2255delinsT; p.(Glu746_Ser752delinsVal), T790M: c.2369C>T; p.(Thr790Met) and L858R: c.2573T>G; p.(Leu858Arg).
