# Supplementary material for: Altered splicing of ATG16‐L1 mediates acquired resistance to tyrosine kinase inhibitors of EGFR by blocking autophagy in non‐small cell lung cancer
Source: Mol Oncol. 2022 Aug 30;16(19):3490–508. doi: 10.1002/1878-0261.13229 (PMC9533692; doi:10.1002/1878-0261.13229)
Supplement: Supplementary file 4 — Table S1. ATG16‐Ex8 status and clinical data of human samples. [file MOL2-16-3490-s005.pdf]

Table S2

gene name	exon	exon regulation	Primer Fw ID	Forward primer	Primer Rv ID	reverse primer
AASDH	2	up	AASDH_ex1_Fw	ATGCGATCAAACCTAATCCG	AASDH_ex3_Rv	GGTGAATCTGGCTCGATAGG
ACOT9	6	up	ACOT9_ex5_Fw	CAGCTTACTCCTGGAAGAGG	ACOT9_ex7_Rv	GTGGATGCTCCTACTATCTC
ADK	7	up	ADK_ex6_Fw	TAGCTAATCTTGCTGCTGCC	ADK_ex9_Rv	TGGGTGAAGATCACGATTCG
ANKHD1	37	down	ANKHD1_ex36_Fw	CTTGCTGATGTTCCAGGAGG	ANKHD1_ex38_Rv	GACTGAAGGAAGCAGACTGG
ARFGAP2	8	up	ARFGAP2_ex7_fw	CCAACACAGACCTGCTTGG	ARFGAP2_ex9_Rv	CTTAGCTGCTGCTGGCTTC
ASXL1	6	up	ASXL1_ex5_Fw	CGCATGCCTCAATGCTATGC	ASXL1_ex7_Rv	GCAGGTTCATACAATGGTGT
ATG16L1	8	up	ATG16L1_ex7_Fw	TGACATTGAGGTCATTGTGG	ATG16L1_ex9_Rv	CAGAACCAGGATGAGTATCC
CBFA2T2	8	up	CBFA2T2_ex7_Fw	GACTCTTGTTCTTGCACTGG	CBFA2T2_ex9_Rv	AGAAGGTGTTCGTGCTGAGC
CD46	13	up	CD46_ex12_Fw	CGTACAGATATCTTCAAAGG	CD46_ex14_Rv	TGATTTAGTCTGGTAAGTGG
CLASP1	22	up	CLASP1_ex21_Fw	ATTGATGTGAACGCAGCAGC	CLASP1_ex24_Rv	TATCAGGTGTAGAGGCGACG
DCAF10	6	up	DCAF10_ex5_Fw	TAGAAGTAGGCAGCTATCCC	DCAF10_ex7_Rv	TTTCCGTGGTCACTCTCACC
DFNB31	4	down	DFNB31_ex3_Fw	TCACCAACCACATCTACACC	DFNB31_ex5_Rv	TCACTTCTAGAATCTGGTCC
DHODH	5	down	DHODH_ex4_Fw	TTTCAGTGGTGGAACACAGG	DHODH_ex6_Rv	CAATGTCCTCCTTATCCTGG
DHX33	2	down	DHX33_ex1_Fw	AGTCTGCCCATCTTCCAAGC	DHX33_ex3_Fw	CACAGCTGTATTTCCGAAGC
DNAJC16	2	down	DNAJC16_ex1_Fw	AACTAACTGGAGCACGGAGC	DNAJC16_ex3_Rv	AACTTGTCTTCTGCTCCAGG
EHBP1	5	up	EHBP1_ex4_Fw	TTGGAAGAGACTGCAGCGTG	EHBP1_ex6_Rv	CACAACAACACCACGATAGG
EIF4H	5	down	EIF4H_ex4_Fw	TCACTTCGTGTGGACATTGC	EIF4H_ex6_Rv	CTGAAATCCATGTTGGATCC
ESYT2	16	up	ESYT2_ex15_Fw	CTGACAAAGACCAAGCCAAC	ESYT2_ex17_Rv	CCAACTGACATCTGGACAAC
FAM48A	5	up	FAM48A_ex4_Fw	TTCACCCCACTTCATCAGCG	FAM48A_ex6_Rv	TTTAGGAGGTCTCTGTCGGG
GK	22	up	GK_ex21_Fw	TGGGTTACAACTCAATCTCC	GK_ex23_Rv	GGAATCCATGAGTTGGTAGG
HHLA3	3	up	HHLA3_ex2_Fw	AGCATTCTTGGATCTCTTGC	HHLA3_ex4_Rv	GGAATGTCATCAGTTAAGGC
LRP8	19	up	LRP8_ex18_Fw	TGAGTGGATACCTGATCTGG	LRP8_ex20_Rv	TCAAGGCTTAATGCCACTCG
LTA4H	21	down	LTA4H_ex20_Fw	CAATGCCACAGACCTGAAGG	LTA4H_ex23_Rv	TCTTGGTAGGTTCGGACAGC
MBNL1	12	up	MBNL1_ex11_Fw	AATACCAACAGGCTCTAGCC	MBNL1_ex13_Rv	TGTGGCAGATGTTGTTGCTG
MFSD9	5	up	MFSD9_ex4_Fw	GGAGCAAGTCCAACAGTTGC	MFSD9_ex6_Rv	AAGTAGAGCCCTTGAGATGG
NRF1	8	down	NRF1_ex7_Fw	GAGCATGATCCTGGAAGACC	NRF1_ex9_Rv	TGGACTACAGTCTGTGATGG
PFKM	15	down	PFKM_ex14_Fw	CATCATCATTGTGGCTGAGG	PFKM_ex16_Rv	ACCTGGACACATTCCATGAG
PPP3CB	16	up	PPP3CB_ex14_Fw	CGAGCAATTGGCAAGATGGC	PPP3CB_ex17_Rv	TCCAAACCCTTTGCCTCTTC
PQLC3	6	up	PQLC3_ex5_Fw	TGGAAGACGAGAGACTCAGG	PQLC3_ex7_Rv	TATAGCGGTCTTCCGGTAGC
R3HDM1	16	up	R3HDM1_ex15_Fw	CTTCAGTGGAATCTCAGTCC	R3HDM1_ex17_Rv	ACAACTGGACTCCCATCTGG
RAB17	4	down	RAB17_ex3_Fw	AAGTCCAGCTTGGCTCTTCG	RAB17_ex5_Rv	GCTGAGGTCCGTCTTGTTGC
RALGPS1	7	down	RALGPS1_ex6_Fw	AACTAGCCAGCTGTGGATGG	RALGPS1_ex8_Rv	TGTCAGCCTGAAGATGGGAG
RMND1	2	down	RMND1_ex1_Fw	GCGCTTCCTTCTTCTCTTCC	RMND1_ex4_Rv	ATGCTGTGCAGTGCATTAGG
RNF8	6	up	RNF8_ex5_Fw	AGGACCTGAAGCAACAGCTG	RNF8_ex7_Rv	TGGAGCTCATTCTCTAGCAC
SCMH1	6	dpwn	SCMH1_ex5_Fw	ACCTATCAGAGTCTGCATCC	SCMH1_ex7_Rv	CTGTAGTGGAGCAGCACAGA
SLC25A25	6	up	SLC25A25_ex5_Fw	CGCAGGAGATCATGCAGTCC	SLC25A25_ex7_Rv	GTAGTCTCTCCACTCGTTCC
SLC3A2	4	down	SLC3A2_ex4_Fw	GCTGGTCTTCAACTCTTGGC	SLC3A2_ex4_Rv	TCATATCCACCTCGGTGTCC
STIL	17	down	STIL_ex16_Fw	GCATGTAATGCAACCCTTGC	STIL_ex18_Rv	CCAGAGATCACTGCTTCTGG
SULF2	20	up	SULF2_ex19_Fw	AGATGGAGGAAGCTATGAGC	SULF2_ex21_Rv	CCTGTGCAGTCAGGTGATGC
TMC6	14	down	TMC6_ex13_Fw	GTACTGGAGGTGTACGTGGC	TMC6_ex15_Rv	ATCAGCTCCAGGACATTCCG
TRAPPC9	6	down	TRAPPC9_ex5_Fw	TGAAGCAGCCAATAGACACC	TRAPPC9_ex7_Rv	CGTCCGATCTCAGTACTGGT
VEZT	7	down	VEZT_ex6_Fw	CAATACTTACAGGATCTGGG	VEZT_ex8_Rv	GCCACTTTTAACAGGATACC
ZNF207	10	up	ZNF207_ex9_Fw	CCAACAGCAACTGTACCTGC	ZNF207_ex11_Rv	GCAGGGAATGTAGGCTTTGG
ZNF250	5	dpwn	ZNF250_ex4_Fw	TGGGAATGTAGTCTCATTGG	ZNF250_ex6_Rv	GCTTAAGACCTGAACTTCAC

Genes for which altered exon skipping in PC9 GR resistant cells compared to PC9 sensitive cells was validated by RT/PCR
