# Supplementary material for: Altered splicing of ATG16‐L1 mediates acquired resistance to tyrosine kinase inhibitors of EGFR by blocking autophagy in non‐small cell lung cancer
Source: Mol Oncol. 2022 Aug 30;16(19):3490–508. doi: 10.1002/1878-0261.13229 (PMC9533692; doi:10.1002/1878-0261.13229)
Supplement: Supplementary file 5 — Table S2. Genes for which altered exon skipping in PC9 GR‐resistant cells compared with PC9‐sensitive cells was validated by RT/PCR. [file MOL2-16-3490-s002.pdf]

Table S3

Events	Pvalue<=0.05 & deltaPSI>=10%	ALL
exon_skipping	65	28728
acceptor	80	52466
donor	32	44342
mutually_exclusive	1	183
multi_exon_skipping	33	18112

Parameters used :	
threshold deltaPSI	0,1
threshold pvalue	0,05
Type of analysis	unpaired

Exon skipping

gene_symbol	exon_skipped	coordinates	flanking exons	deltaPSI	pvalue_corrected_glm
ASXL1	6	20:30959581-30959677	5:7	0,49	2,39E-02
DNAJC16	2	1:15855583-15855767	1:3	0,41	4,89E-03
ANKRD9	2	14:102974978-102975189	1:3	0,31	3,51E-02
ACOT9	6	X:23752458-23752484	5:7	0,31	2,48E-12
PPP3CB	16	10:75199630-75199659	15:17	0,29	4,20E-05
GOLGA4	28	3:37402734-37403712	27:30	0,28	4,09E-02
PQLC3	6	2:11315094-11315135	5:7	0,28	4,63E-02
GK	22	X:30745583-30745669	21:23	0,27	1,19E-02
ODF2	5	9:131219477-131219716	3:6	0,27	4,09E-02
HIPK3	15	11:33369712-33369774	14:16	-0,25	4,61E-04
AASDH	2	4:57250236-57250507	1:3	0,25	4,36E-02
TRAPPC9	6	8:141436714-141436740	5:7	-0,24	3,42E-02
RABL2B	2	22:51221319-51221714	1:3	0,24	3,97E-03
STIL	17	1:47725961-47726211	16:18	-0,24	2,93E-03
FAM48A	5	13:37622701-37622736	4:6	0,24	6,02E-09
SCMH1	6	1:41625397-41625605	5:7	0,23	3,39E-02
ZNF250	5	8:146112225-146112287	4:6	-0,21	3,53E-02
KIAA1712	11	4:175237681-175237718	10:12	-0,21	4,06E-03
PLD3	4	19:40871625-40871837	3:5	0,20	2,94E-03
LAMP2	10	X:119570349-119573148	9:11	0,20	0,00E+00
DHX33	2	17:5366849-5367009	1:3	-0,20	7,90E-03
EHBP1	5	2:62974530-62974587	4:6	0,19	0,00E+00
RALGPS1	7	9:129812322-129812411	6:8	-0,19	1,41E-02
MLPH	14	2:238454100-238454288	13:15	-0,18	1,55E-02
PFKM	15	12:48529074-48529166	14:16	-0,18	3,13E-03
LRP8	19	1:53715052-53715228	18:20	0,18	3,32E-09
DFNB31	4	9:117228547-117228672	3:5	-0,18	3,86E-02
ATG16L1	8	2:234182367-234182423	7:9	0,18	2,31E-02
VEZT	7	12:95650326-95650398	6:8	-0,18	5,51E-09
HHLA3	3	1:70832616-70832667	2:4	0,18	3,58E-02
ANKHD1	37	5:139918944-139919431	36:38	-0,18	9,90E-04
SULF2	20	20:46288143-46288196	19:21	0,18	5,61E-03
TMC6	14	17:76116734-76116913	13:15	-0,17	7,13E-05
CBFA2T2	8	20:32207323-32207412	7:9	0,17	1,71E-02
DCAF10	6	9:37857238-37857348	5:7	0,17	3,39E-02
MBNL1	12	3:152173331-152173366	11:13	0,17	1,32E-02
MFSD9	5	2:103343304-103343433	4:6	0,17	7,65E-03
DOCK1	23	10:128859932-128860040	22:24	0,17	1,38E-02
RMND1	2	6:151766443-151766960	1:4	-0,16	1,36E-04
CLASP1	22	2:122203025-122203072	21:24	0,16	3,46E-02
C1orf86	12	1:2124086-2124414	11:13	-0,15	1,99E-02
SMC5	19	9:72961495-72961565	18:20	0,15	1,54E-07
SLC25A25	6	9:130864359-130864394	5:7	0,15	4,24E-03
R3HDM1	16	2:136399106-136399360	15:17	0,15	2,31E-02
ESYT2	16	7:158545472-158545534	15:17	0,15	0,00E+00
DHODH	5	16:72055023-72055210	4:6	0,14	3,46E-02
EIF4H	5	7:73604577-73604636	4:6	-0,14	1,63E-03
ZNF207	10	17:30693684-30693776	9:11	0,14	2,26E-02
RNF8	6	6:37342396-37342485	5:7	0,14	4,50E-02
NHP2L1	2	22:42078360-42078591	1:3	-0,13	0,00E+00
TTC17	20	11:43467481-43468079	19:21	-0,13	1,63E-02
SGOL1	7	3:20215741-20216547	6:8	-0,13	3,61E-02
TM7SF3	11	12:27132717-27132869	10:13	0,13	8,53E-04
RBM6	42	3:50147812-50147896	41:43	-0,12	1,07E-02
RBM5	17	3:50147812-50147896	16:18	-0,12	1,07E-02
LTA4H	21	12:96397616-96397759	20:22	-0,12	2,65E-03
RAB17	4	2:238486647-238486798	3:5	-0,12	3,72E-03
CFLAR	6	2:202000921-202004600	5:7	-0,12	1,88E-02
ADK	7	10:76285014-76285184	6:8	0,11	2,88E-02
CD46	13	1:207963598-207963690	12:14	0,11	0,00E+00
CAPN7	17	3:15287033-15287178	16:18	-0,11	4,06E-03
NRF1	8	7:129350214-129350411	7:9	-0,10	3,75E-02
MFI2	7	3:196745825-196746672	6:8	0,10	7,65E-03
SLC3A2	4	11:62644256-62644351	3:5	-0,10	4,24E-03
ARFGAP2	8	11:47194261-47194302	7:9	0,10	4,90E-02

Acceptor

gene_symbol	exon	coordinates	lenght	deltaPSI	pvalue_corrected_glm
ZNF207	15	17:30704940-30707975	0	0,35	4,52E-02
ZNF207	15	17:30704940-30707975	-3	-0,35	4,52E-02
CAMTA2	7	17:4885384-4885522	0	0,32	2,46E-03
CEP350	14	1:179993554-179993717	12	-0,25	6,16E-03
CEP350	14	1:179993554-179993717	0	0,25	6,17E-03
BAT4	3	6:31631630-31632357	3	0,24	2,43E-03
LMO7	20	13:76409288-76409475	0	0,24	1,65E-04
LMO7	20	13:76409288-76409475	81	-0,24	1,65E-04
ACIN1	12	14:23536523-23537880	207	-0,23	2,10E-02
BAT4	3	6:31631630-31632357	0	-0,22	2,42E-03
SMARCAD1	22	4:95200074-95200201	0	-0,21	4,88E-02
SDAD1	3	4:76902525-76902698	75	-0,21	5,81E-04
ADPGK	9	15:73043636-73045236	3	0,20	2,88E-04
NCOR2	24	12:124846672-124846843	3	0,20	2,19E-02
NCOR2	24	12:124846672-124846843	0	-0,20	2,19E-02
ADPGK	9	15:73043636-73045236	0	-0,20	4,97E-04
SDAD1	3	4:76902525-76902698	0	0,19	1,06E-03
BRCA1	8	17:41251792-41251897	0	-0,19	1,26E-02
BRCA1	8	17:41251792-41251897	3	0,19	1,26E-02
FUT3	2	19:5846437-5846977	402	-0,18	2,35E-07
E2F3	4	6:20481437-20481656	0	-0,18	7,48E-03
E2F3	4	6:20481437-20481656	18	0,18	7,48E-03
CYB561D2	2	3:50388774-50388991	0	0,18	3,99E-03
CYB561D2	2	3:50388774-50388991	66	-0,18	3,99E-03
C19orf2	12	19:30505794-30506611	0	0,17	2,69E-07
C19orf2	12	19:30505794-30506611	649	-0,17	2,69E-07
FUT3	2	19:5846437-5846977	348	0,16	5,43E-05
PHF10	4	6:170117919-170118002	0	-0,16	1,88E-02
PHF10	4	6:170117919-170118002	6	0,16	1,88E-02
C9orf156	5	9:100673200-100675257	0	0,16	4,55E-03
C9orf156	5	9:100673200-100675257	1905	-0,16	4,55E-03
FAM189B	2	1:155224191-155224247	0	-0,16	2,46E-03
FAM189B	2	1:155224191-155224247	-79	0,16	2,46E-03
BDP1	9	5:70782311-70782454	0	0,16	3,98E-03
BDP1	9	5:70782311-70782454	47	-0,16	3,98E-03
U2AF1L4	3	19:36235527-36235882	243	0,15	6,77E-03
POLR2H	2	3:184080661-184081353	353	-0,15	3,56E-03
IKBKAP	2	9:111693277-111693481	145	-0,15	2,08E-02
CCDC41	14	12:94727258-94727415	0	-0,15	8,24E-03
NCOR2	12	12:124914159-124914252	0	-0,14	3,67E-02
NCOR2	12	12:124914159-124914252	3	0,14	3,67E-02
RNF216	5	7:5778907-5779256	273	0,14	1,26E-02
WSB1	6	17:25634770-25636298	1356	-0,14	3,98E-03
WSB1	6	17:25634770-25636298	0	0,14	3,98E-03
RNF216	5	7:5778907-5779256	0	-0,14	2,93E-02
ILK	2	11:6625410-6625590	46	0,13	1,59E-02
CCDC41	14	12:94727258-94727415	11	0,13	2,19E-02
AC004410.1	6	19:2339068-2339207	-4	-0,13	3,95E-02
AC004410.1	6	19:2339068-2339207	0	0,13	3,95E-02
RTEL1	6	20:62293827-62293980	72	-0,13	4,55E-03
RTEL1	6	20:62293827-62293980	0	0,13	4,55E-03
DOM3Z	5	6:31938120-31938255	0	-0,13	3,14E-02
DOM3Z	5	6:31938120-31938255	100	0,13	3,14E-02
IDUA	8	4:995256-995351	0	0,13	4,09E-02
IDUA	8	4:995256-995351	4	-0,13	4,06E-02
CLCC1	3	1:109492931-109493070	10	0,12	1,31E-03
ILK	2	11:6625410-6625590	0	-0,12	2,82E-02
MIIP	10	1:12091598-12092106	181	-0,12	4,55E-03
MIIP	10	1:12091598-12092106	0	0,12	4,55E-03
TIA1	12	2:70441481-70441626	3	0,12	5,69E-03
TIA1	12	2:70441481-70441626	0	-0,12	5,69E-03
BCAP29	11	7:107258773-107263762	3302	-0,12	1,29E-02
NAT9	3	17:72769715-72769827	0	-0,11	3,55E-03
NAT9	3	17:72769715-72769827	3	0,11	3,55E-03
SMARCA4	35	19:11168931-11169039	3	0,11	4,55E-03
SMARCA4	35	19:11168931-11169039	0	-0,11	4,55E-03
TMPRSS13	12	11:117774370-117774522	33	0,11	2,26E-03
TMPRSS13	12	11:117774370-117774522	0	-0,11	2,26E-03
ZC3H13	16	13:46539415-46539567	0	-0,11	4,79E-02
ZC3H13	16	13:46539415-46539567	3	0,11	4,79E-02
TCF12	21	15:57565228-57565460	0	-0,11	2,92E-02
TCF12	21	15:57565228-57565460	3	0,11	2,92E-02
DPCD	4	10:103347590-103348157	-11	0,11	2,35E-02
U2AF1L4	3	19:36235527-36235882	0	-0,10	3,12E-02
ARFIP2	2	11:6501553-6501693	40	0,10	1,87E-03
ZNF280D	4	15:56999280-56999348	7	0,10	1,84E-02
ZNF280D	4	15:56999280-56999348	0	-0,10	1,84E-02
MORF4L2	5	X:102933427-102933579	0	0,10	0,00E+00
RBM7	4	11:114276428-114276521	0	-0,10	2,88E-02
RBM7	4	11:114276428-114276521	3	0,10	2,88E-02

donnor

gene_symbol	exon	coordinates	lenght	deltaPSI	pvalue_corrected_glm
PPM1J	1	1:113256680-113257950	-918	-0,40	6,77E-03
PPM1J	1	1:113256680-113257950	0	0,40	6,77E-03
PLXNA3	20	X:153695888-153696031	0	0,34	7,21E-09
PLXNA3	20	X:153695888-153696031	-8	-0,34	7,21E-09
LIG1	24	19:48623424-48624579	-1003	-0,31	1,59E-08
LIG1	24	19:48623424-48624579	0	0,31	1,59E-08
C4orf36	7	4:87813336-87813575	-27	0,31	2,51E-02
CCDC84	6	11:118881931-118882021	0	0,25	3,38E-02
CCDC84	6	11:118881931-118882021	-28	-0,25	3,38E-02
GCNT2	6	6:10528864-10530069	-995	-0,23	5,80E-03
GCNT2	6	6:10528864-10530069	0	0,23	5,80E-03
TSPAN4	1	11:842824-843050	-135	-0,22	9,52E-03
PTGES2	2	9:130889718-130890474	-624	0,19	6,18E-04
WDR4	4	21:44293661-44293801	-52	0,16	8,42E-04
WDR4	4	21:44293661-44293801	0	-0,16	8,42E-04
ZNF76	2	6:35227275-35227618	0	0,15	2,56E-02
ZNF76	2	6:35227275-35227618	-114	-0,15	2,56E-02
C8orf41	1	8:33370600-33370703	122	-0,15	4,44E-02
C8orf41	1	8:33370600-33370703	0	0,15	4,46E-02
DRAM2	2	1:111682123-111682333	0	-0,14	8,59E-04
HNRNPU	1	1:245026915-245027827	-61	-0,13	5,29E-04
HNRNPU	1	1:245026915-245027827	-4	0,13	5,29E-04
ZNF673	1	X:46306624-46306781	0	0,12	3,71E-02
NSUN6	5	10:18903389-18903542	12	-0,11	3,62E-02
NSUN6	5	10:18903389-18903542	0	0,11	3,63E-02
NFATC2IP	2	16:28962258-28962719	0	0,11	4,58E-02
NFATC2IP	2	16:28962258-28962719	-347	-0,11	4,58E-02
GRHL2	1	8:102504666-102505077	0	-0,11	3,77E-03
CUTA	1	6:33385736-33386065	90	0,11	9,85E-12
PHC2	12	1:33799691-33799898	0	0,11	1,32E-03
PHC2	12	1:33799691-33799898	-95	-0,11	1,32E-03
POLL	1	10:103347961-103348027	382	0,11	4,18E-02

Mutually exclusive

gene_symbol	exon_skipped	coordinates	flanking exons	deltaPSI	pvalue_corrected_glm
AKR1C2	2:3	10:5049599-5049698;10:5045944-5046206	1:4	-0,34	0,00E+00

Multi exon skipping

gene_symbol	exons_skipped	coordinates	flanking exons	deltaPSI	pvalue_corrected_glm
NUMA1	5:6	11:71773635-71773732;11:71752005-71752132	4:7	0,50	2,40E-04
MAGED2	2:3	X:54834795-54834866;X:54835493-54835656	1:4	-0,39	0,00E+00
GALNS	2:4:5	16:88922615-88922709;16:88909438-88909607;16:88909114-88909237	1:6	-0,37	4,36E-03
EPB41L1	7:8:9	20:34713345-34713449;20:34742662-34742818;20:34761686-34761876	6:10	0,33	4,97E-02
ATP6V0E2	4:5	7:149575767-149575879;7:149576255-149576314	2:6	0,32	4,97E-02
XRCC5	11:12:13	2:217001811-217001948;2:217002812-217002902;2:217005909-217006042	10:14	0,31	0,00E+00
STRA6	3:4:5:6	15:74500038-74500395;15:74495344-74495559;15:74495054-74495220;15:74494759-74494867	2:7	-0,30	1,22E-04
GRB10	4:5	7:50848324-50848433;7:50823584-50823753	2:6	-0,27	2,55E-02
STRA6	2:3:4:5:6	15:74501141-74501371;15:74500038-74500395;15:74495344-74495559;15:74495054-74495220;15:74494759-74494867	1:7	-0,26	7,44E-04
AKR1C2	2:3	10:5049599-5049698;10:5045944-5046206	1:4	-0,26	0,00E+00
UIMC1	9:10	5:176396598-176396707;5:176395556-176396292	8:11	-0,25	1,24E-06
SLC12A9	4:5	7:100452242-100452376;7:100453328-100453934	3:6	0,22	3,31E-02
MFF	8:9:10	2:228207461-228207535;2:228211942-228212100;2:228217230-228217289	7:11	0,21	2,33E-02
XIAP	2:3	X:122994045-122994143;X:123019481-123020389	1:4	0,20	4,00E-03
UNK	3:4:5	17:73787841-73788100;17:73788394-73788559;17:73789524-73790277	2:6	0,18	6,49E-03
NUDT2	2:3	9:34336227-34336339;9:34338711-34338845	1:4	0,17	1,69E-02
TMEM87A	2:3	15:42565087-42565265;15:42564261-42564321	1:4	0,17	3,59E-03
TBC1D19	5:6:7:8:9	4:26638833-26638907;4:26640393-26640456;4:26641763-26641809;4:26661219-26661329;4:26667955-26668027	4:10	0,17	3,82E-02
CEP110	30:31	9:123920018-123920158;9:123920261-123920387	29:32	-0,17	4,83E-03
TNFRSF1A	7:8	12:6440406-6441159;12:6440019-6440092	6:9	-0,15	4,09E-03
PHLDB2	6:7	3:111578027-111578423;3:111578568-111578725	5:8	-0,15	1,59E-03
MAPK12	15:16	22:50691331-50691909;22:50689403-50689834	14:17	-0,14	2,88E-02
ERBB2IP	22:23:24	5:65364705-65364848;5:65367997-65368119;5:65370852-65371058	21:25	0,14	2,78E-02
ABHD11	5:6	7:73151551-73151721;7:73151259-73151440	4:7	-0,14	4,00E-03
LRRC8A	2:3	9:131644781-131645188;9:131648294-131648400	1:4	0,14	3,22E-02
OPA1	4:5	3:193334967-193335074;3:193335573-193335626	3:6	0,14	1,37E-02
ANKRD11	8:9	16:89365284-89367370;16:89358089-89358185	7:10	-0,13	5,69E-03
DHRS4	5:6:7	14:24434971-24435041;14:24435141-24435192;14:24435492-24435626	4:8	0,12	4,00E-03
DPH5	2:3	1:101490865-101491022;1:101488491-101488885	1:4	0,11	4,34E-02
MAP4K4	17:18	2:102477287-102477448;2:102480283-102480513	16:19	0,11	5,90E-03
SEC31A	3:4	4:83820866-83820890;4:83819142-83819215	2:5	0,11	2,75E-02
MRPS6	2:3	21:35467162-35478561;21:35497641-35497780	1:4	-0,11	3,09E-03
MUC4	9:10	3:195497549-195497757;3:195497087-195497242	8:11	-0,11	4,44E-02
