# Supplementary material for: Altered splicing of ATG16‐L1 mediates acquired resistance to tyrosine kinase inhibitors of EGFR by blocking autophagy in non‐small cell lung cancer
Source: Mol Oncol. 2022 Aug 30;16(19):3490–508. doi: 10.1002/1878-0261.13229 (PMC9533692; doi:10.1002/1878-0261.13229)
Supplement: Supplementary file 6 — Table S3. Altered splicing events in PC9 GR‐resistant cells compared with PC9‐sensitive cells. [file MOL2-16-3490-s003.pdf]

Figure S1

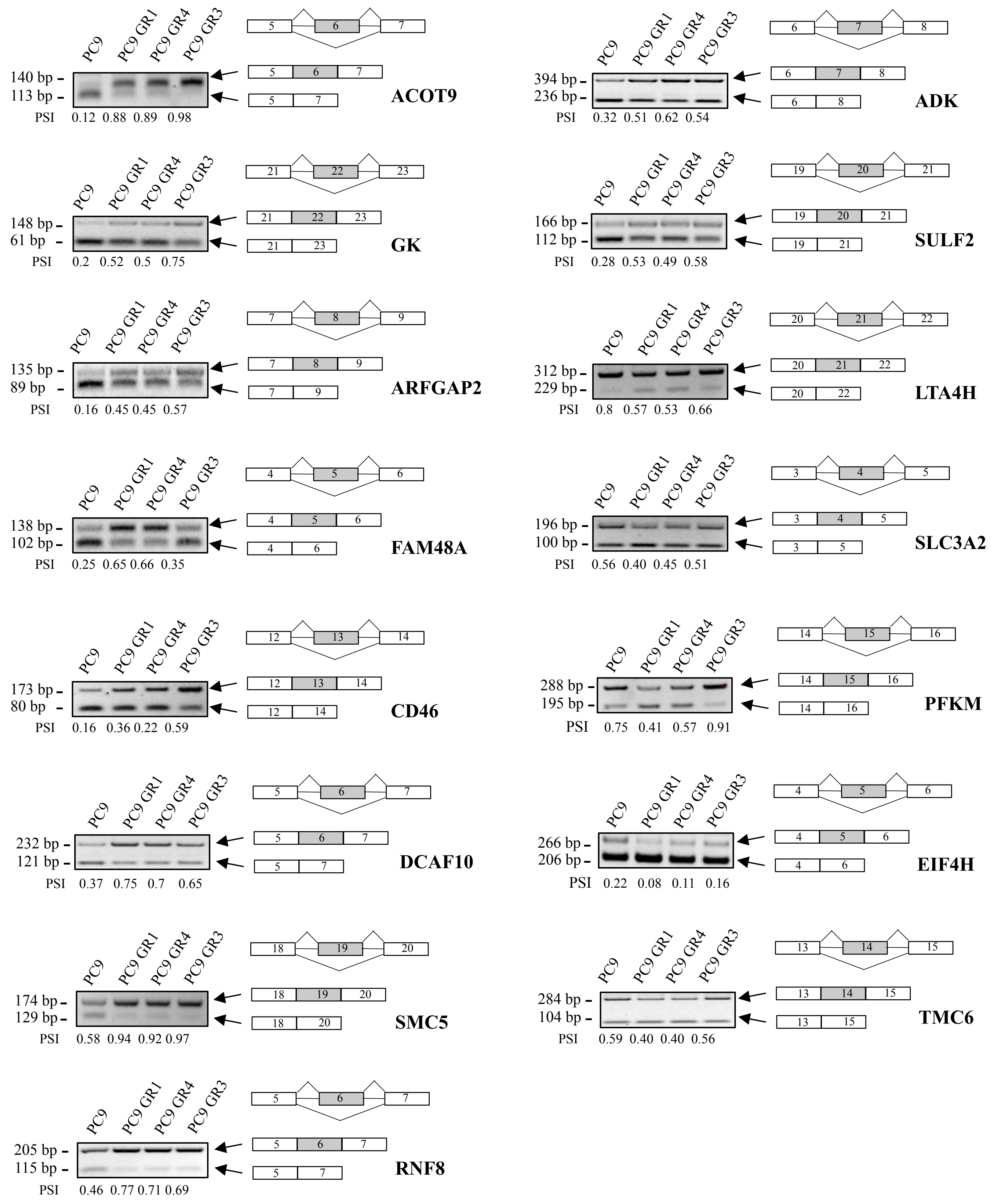

Validation of RNA seq data. RT/PCR analysis of representative examples of altered exon skipping events (in grey) showing inclusion (ACOT9, GK, ARFGAP2, FAM48A, CD46, DCAF10, SMC5, RNF8, ADK, SULF2,) or exclusion (LTA4H, SLC3A2, PFKM, EIF4H, TMC6) of exons in resistant clones (PC9 GR1/3/4) compared to sensitive cells (PC9). Percent Spliced In (PSI) is indicated below the PCR blots.
